# SpliceDetector: a software for detection of alternative splicing events in human and model organisms directly from transcript IDs

**DOI:** 10.1038/s41598-018-23245-1

**Published:** 2018-03-22

**Authors:** Mandana Baharlou Houreh, Payam Ghorbani Kalkhajeh, Ali Niazi, Faezeh Ebrahimi, Esmaeil Ebrahimie

**Affiliations:** 1 Institute of Biotechnology, Shiraz University, Shiraz, Iran; 2 Science and Research Branch, Islamic Azad university, Hamedan, Iran; 3 Department of Biology, University of Qom, Qom, Iran; 4 Adelaide Medical School, The University of Adelaide, Adelaide, Australia; 5 School of Information Technology and Mathematical Sciences, Division of Information Technology, Engineering and the Environment, The University of South Australia, Adelaide, SA Australia; 6 School of Biological Sciences, Faculty of Science and Engineering, Flinders University, Adelaide, SA Australia

## Abstract

In eukaryotes, different combinations of exons lead to multiple transcripts with various functions in protein level, in a process called alternative splicing (AS). Unfolding the complexity of functional genomics through genome-wide profiling of AS and determining the altered ultimate products provide new insights for better understanding of many biological processes, disease progress as well as drug development programs to target harmful splicing variants. The current available tools of alternative splicing work with raw data and include heavy computation. In particular, there is a shortcoming in tools to discover AS events directly from transcripts. Here, we developed a Windows-based user-friendly tool for identifying AS events from transcripts without the need to any advanced computer skill or database download. Meanwhile, due to online working mode, our application employs the updated SpliceGraphs without the need to any resource updating. First, SpliceGraph forms based on the frequency of active splice sites in pre-mRNA. Then, the presented approach compares query transcript exons to SpliceGraph exons. The tool provides the possibility of statistical analysis of AS events as well as AS visualization compared to SpliceGraph. The developed application works for transcript sets in human and model organisms.

## Introduction

Transcripts are products of pre-mRNA splicing processes. Novel transcripts discover each day^1,2^ and add to public databases. Development of high throughput transcriptome sequencing (RNA-seq) has provided a new opportunity to thoroughly investigate the expression differences between genes as well as within the transcripts of a gene^3^. Compared to microarrays, RNA-seq technology allows higher accuracy in discovery of splice junctions and sequences^4^. AS event and its types are important in composition of protein domains, drug designing and drug resistance^5,6^.

In the splicing process, introns are removed from pre-mRNA, and exons fit together with various arrangements. Consequently, each gene develops distinct transcripts to produce distinct proteins. Depending on the AS pattern, properties of cell construction, functions or destination may be affected. It has been revealed that many diseases are associated with the change of particular AS pattern in transcripts^5,7,8^, such as spinal muscular atrophy (SMA) disease^9^ and Hutchinson-Gilford progeria syndrome (HGPS)^10^.

**Figure 1:**
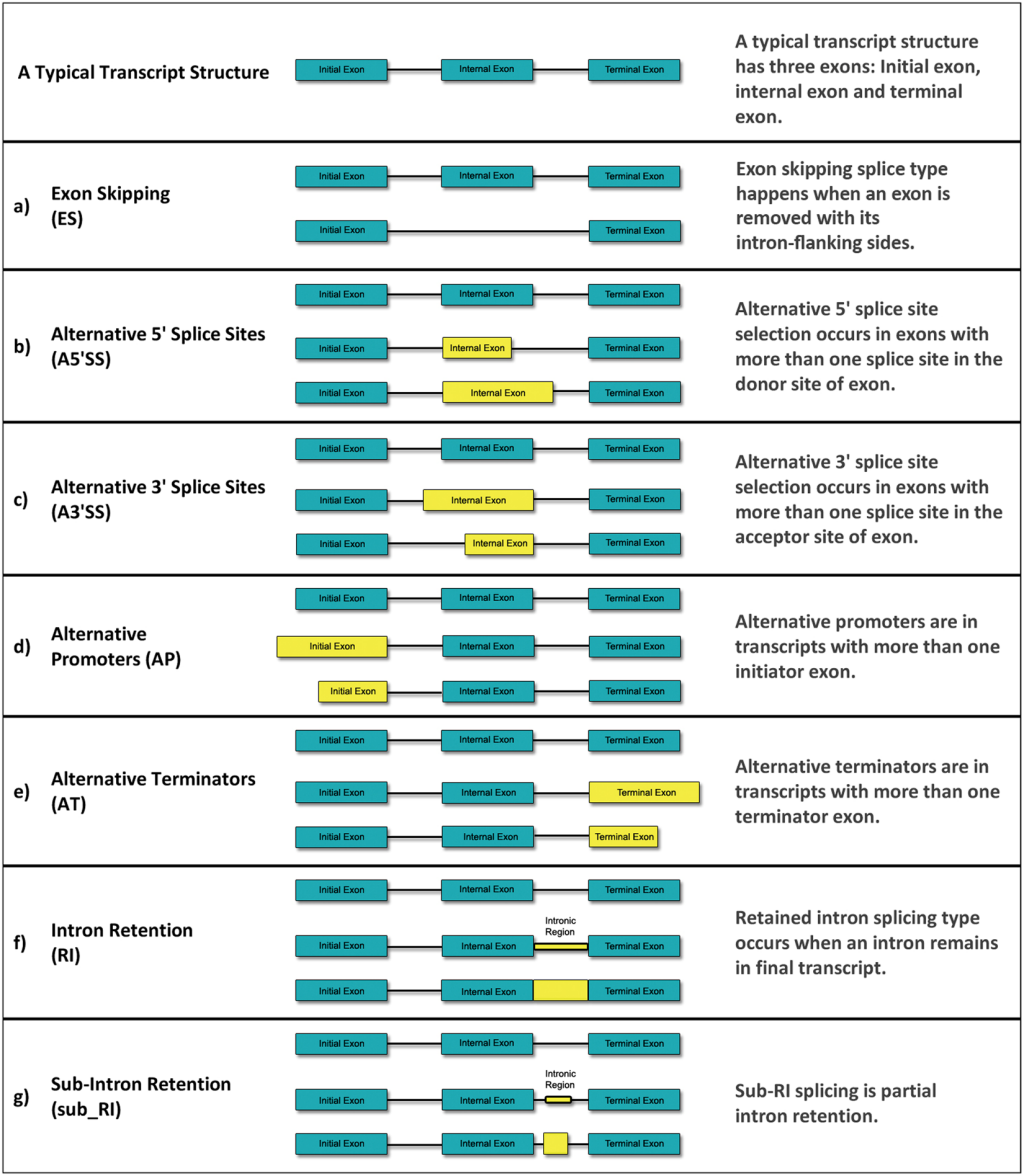
Different types of Alternative splicing (AS) events. (**a**) Exon skipping splice type happens when an exon is removed with its intron-flanking sides. (**b** and **c**) Alternative 5′ splice site and alternative 3′ splice site selections are the splicing types of exons with more than one splice site at one end of an exon. If both ends of an exon are alternate splice sites, the alternate 5′ and 3′ splice site selection occurs. (**d** and **e**) Alternative promoters and alternative terminators are in transcripts with more than one initiator or terminator exon. (**f**) Retained intron splicing type occurs when an intron remains in final transcript. (**g**) Sub-RI splicing is partial intron retention.

Various types of AS events are known and are divided into 5 groups (Fig. 1). The first one is exon skipping (ES) where an exon is removed together with its introns on both sides of the transcript. The second and third types of alternative splicing are related to both the 3′ and 5′ ends of exons(A5′ss & A3′ss). These types of AS events occur when there are more than one splice site at one end of an exon. If an exon has both of these splicing sites, the alternative 5 and 3 splicing sites will be formed (A5′ & 3′ss). The fourth type is introns retaining (RI) where introns remain in transcript. This is the rarest known type in both vertebrates and invertebrates (less than 5 percent of AS events). There is another type of splicing type related to the latter type which includes a partial retention of an intron. We call it sub_RI type. The last group of AS events takes place when first or last exon or both of them are alternates of first or last putative exons and make alternate promoters and alternate terminators splicing types^11,12^.

Transcripts are the important output of many high throughput transcriptome analysis tools which are widely used in RNA-seq data analysis^13^. However, many of the AS finding tools do not have the sufficiency of finding AS events straightly from specified transcripts.

There is an increased attention to develop tools to extract and analyze AS events. A majority of these tools implement AS analysis using transcripts reconstruction. In some tools, performing alignment with a reference genome for model organisms is the basis of analysis. For instance, SpliceSeq works based on known splice junctions and detects AS events using inclusion of exons and splice junctions in transcripts^14^. Another tool, Cufflinks/Cuffdiff gets a prerequisite data in GTF format as reference for comparison^15,16^ and works based on alignment approach. The second category of AS discovery tools reconstructs transcripts without any reference. Trinity methodology for *de novo* full-length transcriptome reconstruction^17^ and ASGS which knows alternative splicing junction though the approach of SpliceGraph forming^18^ are within this category. A more complete list has been offered in supplementary materials (Supplemental files, S1). In addition, most of these applications and web tools need a high level of computer skills and also a prerequisite data for their data processing tasks^19–28^. To work with current tools, it is a necessity for the researchers to be familiar with data formats and software environments.

There is a need for new tools with the capability of directly AS occurrence analysis in a set of transcripts. In order to fill the mentioned gap, our application was designed to discover AS events from known transcripts at a high speed and in a simple and user friendly environment. The developed application in this study solves the above mentioned problems and has considerable advantages. The software does not need any computer skill. Furthermore, the need to data updating was eliminated by using the updated information placed in the Ensembl database to form SpliceGraphs. The basic pathway of the application includes, taking transcript IDs as input, building a SpliceGraph based on all of the exon coordinates of the related gene, and producing AS events as output.

## Methods

### Application Architecture and Data Acquisition

**Figure 2:**
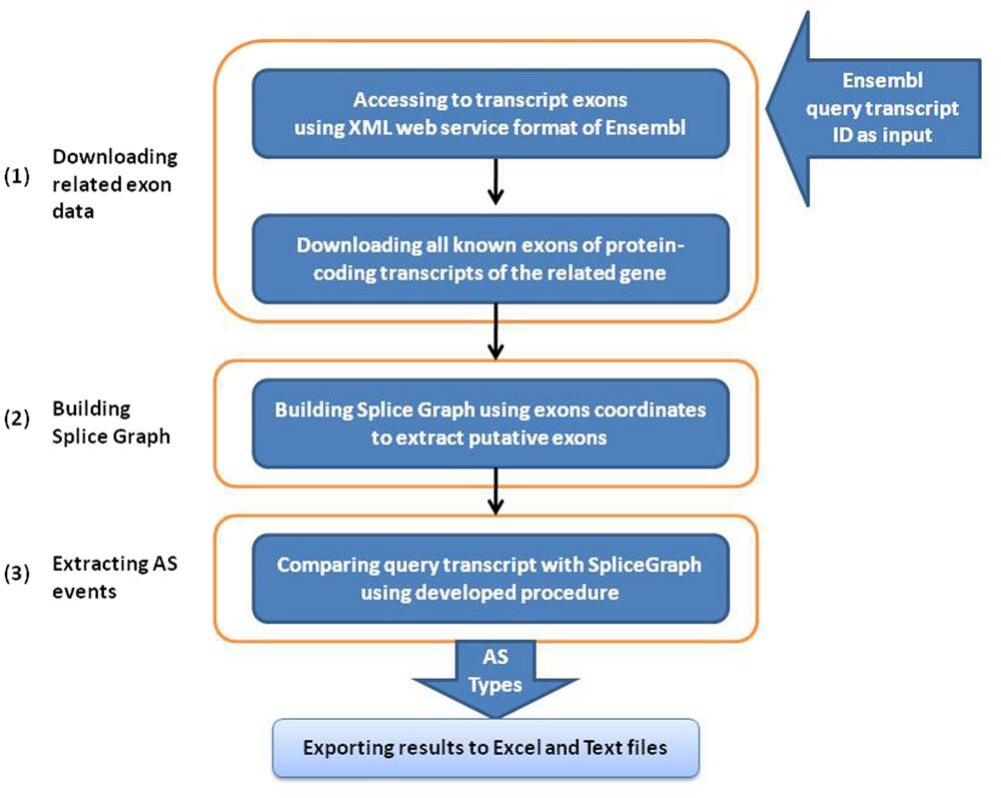
Fellow chart of the employed strategy in this study for extracting the Alternative Splicing events of transcript IDs through building a SpliceGraph. In the first phase, query transcript exons and all known exons of protein-coding transcripts of the related gene is downloaded using XML web service format of Ensembl. In the next phase, exons which do not follow the SpliceGraph construction rules is eliminated and consequently, SpliceGraph is built by remained exons coordinates, and in the last phase, query transcript exons are compared with SpliceGraph exons to extract alternative splicing types.

The application has been coded in Microsoft Visual Studio utilizing C#. NET and comprises two main parts: the SpliceGraph builder and AS events finding. Due to the open source software and the relational database system of the Ensembl database^29,30^, we used Ensembl database to obtain the required data for building SpliceGraph and extracting AS events of known transcripts. The protein-coding type of transcripts was applied as the resource transcripts for software. These basic processes are depicted in Fig. 2.

#### SpliceGraph building

**Figure 3:**
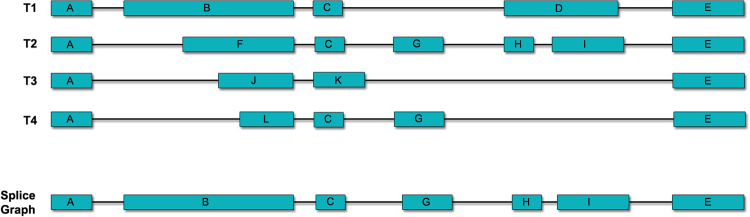
Classification of exons into putative and alternative types. In the first phase, exons A, C, G, and E are selected as a putative exons based on the highest frequency of their splice sites. In the second phase, we focused on exon length. This means that we selected putative exons by their nucleotide numbers when we had an equal frequency of splice sites. Thus, exon B was selected as putative exon. In the next phase, if an exon was equivalent to several smaller exons, we selected multiple exons as putative exons. Therefore, exons H and I were selected as putative exons.

Building SpliceGraphs is the basic part of many splicing detector tools^14,18,31,32^. To ease the difficulty of case by case analyzing of each splice variant and also to investigate the relationship between different transcripts, the approach of graph representation of splicing variants was employed^33^. Graphs include putative exons to use for comparing and extracting AS. Various tools use different methods to build SpliceGraphs. SpliceGrapher^32^ as a Python-based scripting tool constructs the SpliceGraph by summarizing short reads aligned to a reference genome. SplAdder^34^ integrates annotation information and RNA-Seq data to generate an augmented splicing graph, and SpliceSeq^14^ summarizes known transcript variations and knowledge about gene structure into a directed acyclic graph. Requiring a prerequisite data as a reference data is a noticeable clue in all of these mentioned tools. However, our approach has been established on the frequency of active splice sites in pre-mRNA which is provided by the SpliceDetector application directly from Ensembl database due to online mode of software. In the first step, exons with the highest frequency of their splice sites were selected as putative exons. Then, the lengths of exons were considered as the selection factor and longer exons were selected as putative exons when we had an equal frequency of splice sites. In the third step, we selected multiple exons as putative exons when an exon was equivalent to several smaller exons. Figure 3 shows the rules applied in this project for building SpliceGraphs.

### Rules applied in SpliceGraph building

- In the first phase, putative exons were selected based on the highest frequency of the splice sites which are known as the exons start and end points.
- In the second phase, the lengths of exons were considered. It means, putative exons were selected regarding to their nucleotide numbers when there was equal frequency of splice sites. In other words, minimum start point for repeated end points and maximum end point for repeated start points were selected.
- At the third step, multiple exons were selected as putative exons, if an exon was equivalent to several smaller exons. In other words, when an exon in a transcript includes some shorter exons in another transcript, the multiple exons were classified as putative exons.

The gene in the example has 4 transcripts and Fig. 3 shows how these rules of classification have been applied.

### Steps to form SpliceGraph

1. Using BioMart of Ensembl database and the XML web service format, all known exons of protein-coding transcripts of the related gene were downloaded. The obtained exon set might have duplicated exons.
2. Reverse strand transcripts, presented as the minus strand direction in the downloaded data, were turned over using their genomic positions to be considered as forward strands.
3. All start and end points of all exons were collected in a pool, regardless of exon repetition, transcript length and transcripts support level (http://www.ensembl.org/Help/Glossary).
4. The collected start and end points of mentioned exons were sorted and their frequencies were measured.
5. Putative exons were selected using the previously mentioned rules regarding their start or end properties and then the SpliceGraph was formed.

As an example, we present the steps of SpliceGraph building for an Ensembl transcript ID of *OSGIN*1 gene.

Example Query Transcript ID:ENST00000565123.

**Retrieving required data:**

At the first step, genomic coordinates of query transcript ID was downloaded using an XML file (Supplemental files, S3) to retrieve genomic coordinates of query transcript exons. In order to apply an integrated approach for all transcripts, downloaded coordinates of reverse strand transcripts were turned over to form forward strand coordinates for reverse transcripts. Then, all exon coordinates of the gene of interest were downloaded using retrieved gene ID.

**Algorithm implementation:**

Our designed algorithm employed GROUP BY clause to measure the frequency of all retrieved start and end points of all exons which are collected in a digits pool.

**SpliceGraph formation:**

For SpliceGraph building, putative exons were selected using the designed rules regarding their start and end properties. By eliminating the exons that do not follow the SpliceGraph construction rules, we have a SpliceGraph including 8 putative exons(Supplemental Tables, ST1–5).

#### Method of comparison

We designed an integrated algorithm to compare the query transcript exons with the SpliceGraph exons. The algorithm takes the start and end coordinates of the query transcript and the relevant arranged SpliceGraph coordinates as input and gives splice types as output. SpliceDetector source code is available in the supplemental files (S2).

### The algorithm of data processing

If E_1_T is the first exon of the query transcript and E_1_G is the first exon of the s SpliceGraph that has been built using the query transcript, we have:
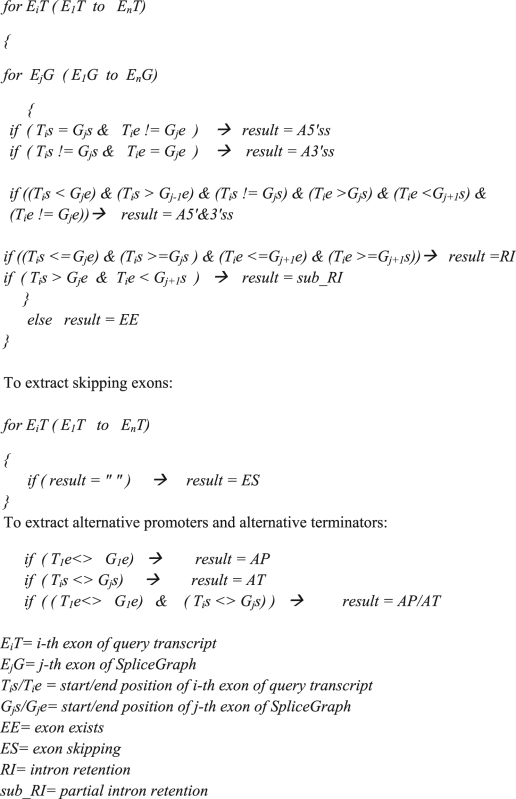


### Differential splicing analysis

In addition to expanding proteome diversity, alternative splicing may produce splice forms that are not translated into proteins, but play major roles in regulation of gene expression^35^. In order to study the effect of treatment on AS events alteration before and after the treatment, we added a statistical analysis of AS events of transcripts to our software. We considered unique mapped transcript reads as effective read count for AS events to avoid read mapping errors and prevent false positive outcomes^36^. In a comparison between an experimental group and a control group, the number of AS events of each transcript before treatment can be calculated by AS events number of that transcript multiply by its unique reads count in control sample (before treatment);

*Total ES events count for each transcript before the treatment* = Unique transcript mapped reads count before treatment * ES event number of the transcript in control sample.

Similarly, the number of AS events of each transcript after treatment can be calculated by AS events number of that transcript multiply by its unique mapped reads count in treated sample (after treatment).

*Total ES events for each transcript after the treatment* = Unique transcript mapped reads count after treatment * ES event number of the transcript in treated sample.

Regarding the fact that transcripts without differential expression have the same amount of AS events and expression rates before and after the treatment, we can get an estimation of AS events changes using AS events of differentially expressed (DE) transcripts under the treatment. The Chi-square goodness-of-fit test is used for nominal variables and calculates the probability of getting a result like observed data under the null hypothesis^37^. Therefore, we applied the Chi-Square goodness of fit test to compare AS events abundance before and after the treatment. Treatments may alter the amount of AS events in each transcript and differentially expressed transcripts usually exhibits a significant alteration in the number of AS events before and after the treatment due to their significant different expression. The presented comparison approach examines the overall changes in the amounts of AS events. Table 1 shows a simplified example for ES (exon skipping) event.

**Table 1:** An example of comparing Alternative Splicing events abundance before and after treatment. Total number of Exon Skipping events for each transcript before the treatment equals with Unique transcript number of reads before treatment multiply by ES event number of that transcript in control sample and similarly, total number of ES events for each transcript after the treatment equals with Unique transcript number of reads after treatment multiply by ES event number of that transcript in treated sample. Then a Chi-square goodness of fit test evaluates the significance of the difference in total number of ES events on the whole experiment level before and after the treatment. The number of final events may be adjusted on the whole experiment level with a non significant p-value (part a), or show a significant total alteration of AS events (part b).

	transcripts	Fold Change	Exon Skipping Event count	Before treatment (Control)		After treatment (Treatment)	
				Unique reads count	All transcripts ES event	Unique reads count	All transcripts ES event
(**a**)	Transcript 1 (Upregulated)	2	1	40	40	80	80
	Transcript 2 (Downregulated)	0.5	2	40	80	20	40
	Total ES event				**120**		**120**
	Chi square goodness of fit (120,120): 1 → p-value: Not significant						
(**b**)	Transcript 1 (Upregulated)	2	2	40	80	80	160
	Transcript 2 (Downregulated)	0.5	1	40	40	20	20
	Total ES event				**120**		**180**
	Chi square goodness of fit (120,180): 0.0005 → p-value: Significant						

**Figure 4:**
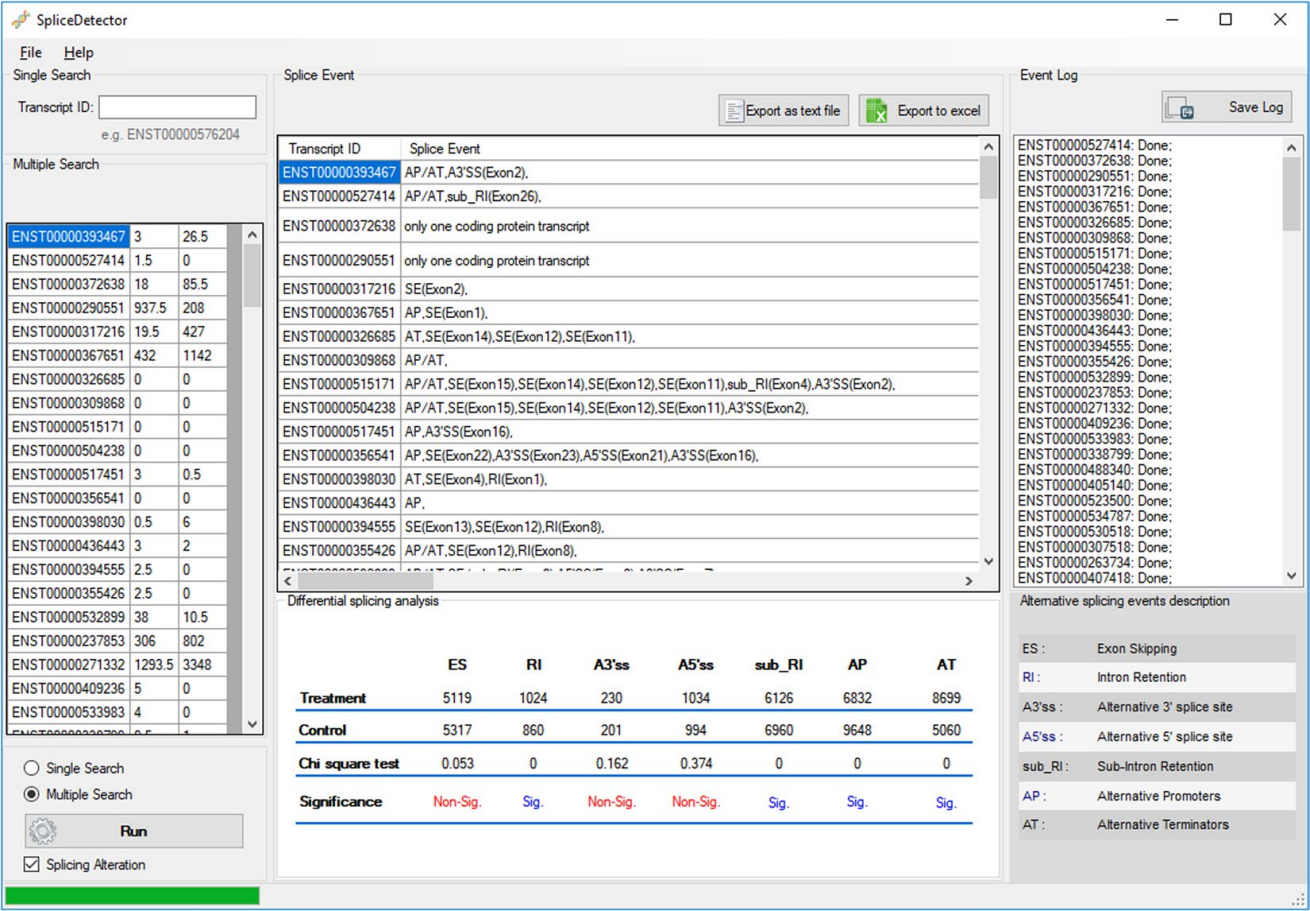
Statistical analysis of splicing events alteration in splicing variants before and after the treatment. The application performs a Chi-square Goodness of Fit statistical test to calculate significance of alteration rates between the Experimental Group and Control Group using the estimated number of alternative splicing events.

As an example, we performed a statistical analysis of AS events in a set of DE transcripts upon treatment with Genistein (the soy isoflavone metabolite). This DE transcripts list was generated from MCF-7 breast cancer cell line RNA-Seq data (FASTQ files) downloaded from GEO database under accession number GSE56066^38^. Figure 4 shows the outcome of applied statistical test on the transcripts associated with ‘transcription’ gene ontology. According to results of the test (Table 2), AS events including RI (Retained Intron), sub_RI (sub-Retained Intron), AP (Alternative promoter) and AT (Alternative Terminator) event types exhibited significant differences in occurrence between control and treated samples. In contrast, ES (Exon Skipping), A3′SS (Alternative 3′ splice site) and A5′SS (Alternative 5′ splice site) event types did not show a significant difference. The data related to DE transcripts identification and gene ontology analysis can be viewed in in the supplemental files (S4).

**Table 2:** Results of performed statistical analysis of AS events in MCF-7 breast cancer cell line after treatment with Genistein. The input data was the differentially expressed transcripts, associated with ‘transcription’ gene ontology, of MCF-7 breast cancer cell line under GEO accession number GSE56066^38^. According to results of the test, AS events including RI (Retained Intron), sub_RI (sub-Retained Intron), AP (Alternative promoter) and AT (Alternative Terminator) event types exhibited significant differences between control and treated samples and ES (Exon Skipping), A3′SS (Alternative 3′ splice site) and A5′SS (Alternative 5′ splice site) event types did not show a significant differences.

	Total count of splice events						
	Exon Skipping (ES)	Retained Intron (RI)	Alternative 3′ splice site (A3′SS)	Alternative 5′ splice site (A5′SS)	sub-Retained Intron (sub_RI)	Alternative promoter (AP)	Alternative Terminator (AT)
Treated sample	5119	1024	230	1034	6126	6832	8699
Control sample	5317	860	201	994	6960	9648	5060
Chi Square value	0.053	0	0.162	0.374	0	0	0
p value significance	Not- significant	Significant	Not- significant	Not- significant	Significant	Significant	Significant

## Results

**Figure 5:**
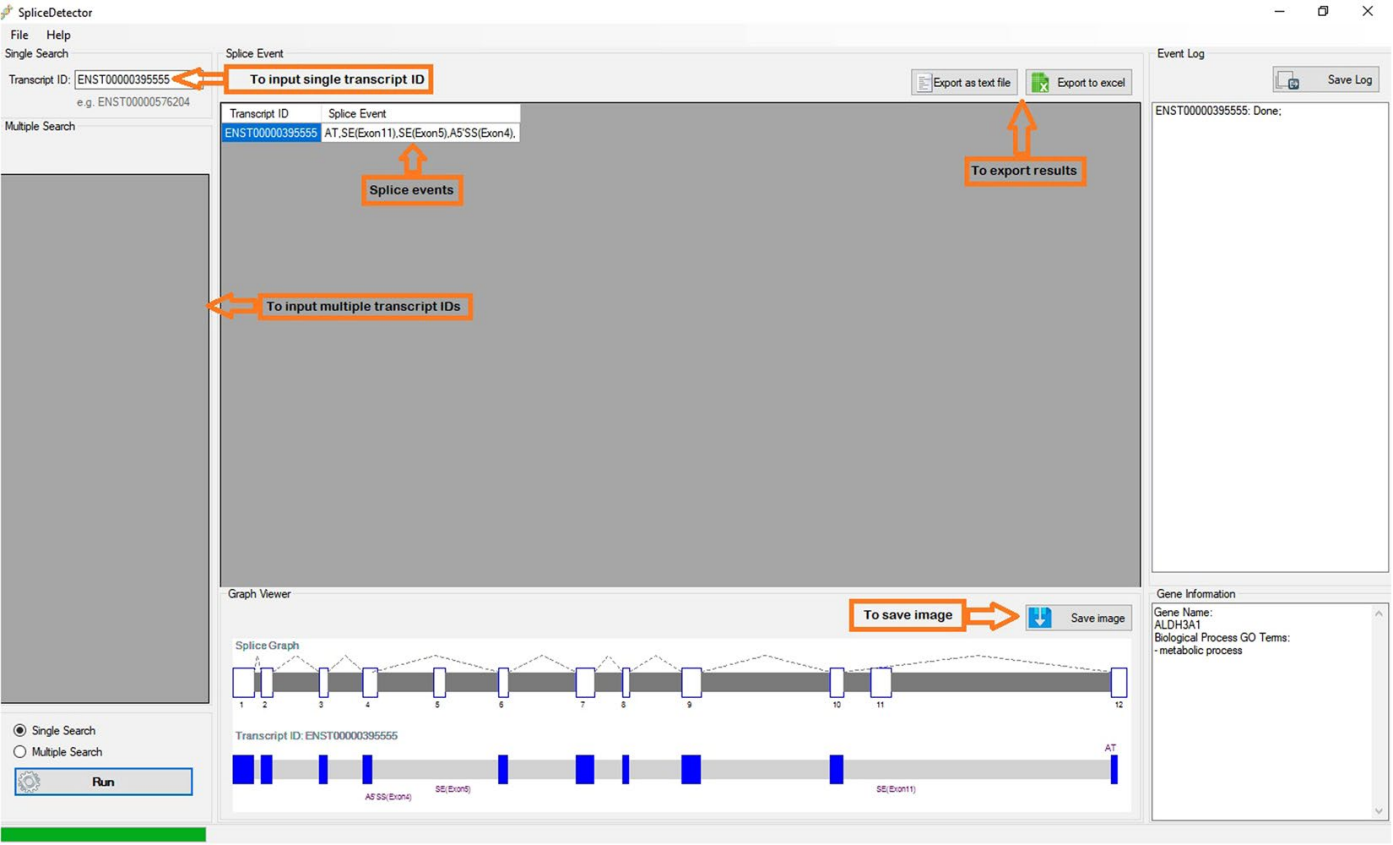
The input and output of SpliceDetector Software. Application accepts transcript IDs as input in both single and multiple forms. Results are exportable in excel and text format.

Unlike the other AS detector tools, our application detects AS events types directly from transcripts without any advanced computer skills, prerequisite application installation, or required data downloading by users. The application works in two forms of single and multiple forms (Fig. 5) and accepts the query transcript IDs in Microsoft office excel, GTF, and GFF3 formats. A graph which represents the query transcript exons as well as the constructed SpliceGraph, illustrates the alternative splicing regions and offers an understanding of splice sites and alternative splicing events.The online working mode of the application results in low application size. Furthermore, due to the downloaded references from Ensembl site, SpliceGraph are updated in each use. Meanwhile, this eliminates the need for application updating or the need to any given repository or database data and reference.

### Data Storage, Visualization and Updating

The present application does not require any given (repository or database) data. The only requirement for application installation on private computers is. NET Framework 4.5 (or higher) and the only given data is transcript IDs of interest. In addition, this tool works online (connected to the Internet), so, SpliceGraph building process relies on updated data of Ensembl database and there is no need for the users to get involved. This application is not specific to a particular organism and works with all model organisms on Ensembl database.

In order to examine the results of SpliceDetector application, we downloaded the result obtained from an experiment made by Obstetrics and Gynecology department of University of Alabama at Birmingham in 2014 where ovarian cancerous tissue was treated with the herbal drug paclitaxel (PTX) derived from a plant called *Taxusbrevifolia* (Pacific yew)^39^. We implemented RNA-seq analysis on downloaded short reads to get their known transcripts based on Ensembl database using CLC Genomic Workbench 9.0.0 software (https://www.qiagenbioinformatics.com). In order to get differential expression details, the proportions-based (Baggerley’s) test was applied on results. We filtered result data based on p-value less than 0.01 and a fold change more than 2.5 in treated samples against controls (Supplemental files, S5). Two of the differentially expressed genes which we found were *TMEM123* (Transmembrane Protein 123) and *DHRS4L2* (Dehydrogenase/Reductase 4 Like 2). The ENST00000361236 transcript of the *TMEM123* gene has been downregulated and the ENST00000335125 transcript of the *DHRS4L2* gene has been upregulated due to the treatment. These alterations are originated from changes in AS events patterns occurring in transcripts formation. Therefore, we can extract each transcript splicing type and compare them. Below is the results of SpliceDetector application analyzing.

ENST00000361236: AT,SE(Exon5),SE(Exon4)

ENST00000335125: AP,RI(Exon9),RI(Exon7)

These results show an alteration in exon skipping of exons 4 and 5 in *TMEM123* under paclitaxel. In contrast the treatment increases the retention of the introns 7 and 9 in *DHRS4L2*. Investigating the gene ontology analysis of *TMEM123* gene, through the Ensembl gene ontology annotation led us to necrotic cell death while the *DHRS4L2* involves in oxidation-reduction process that results in the removal or addition of one or more electrons to/or from a substance.

### Verifying the results of the application

Regarding the lack of an application or webtool with similar operation to our SpliceDetector application, we decided to verify output of our software with Ensembl splice variants through manual checking. We selected *APOA2* gene with ENSG00000158874 Ensembl gene ID. According to GeneCards database (http://www.genecards.org)^40^ information, *APOA2* gene encodes apolipoprotein (apo−) A-II, as the second most abundant protein of the high density lipoprotein particles. *APOA2* is associated with Hypercholesterolemia, Familial and Aapoaii Amyloidosis.

**Figure 6:**
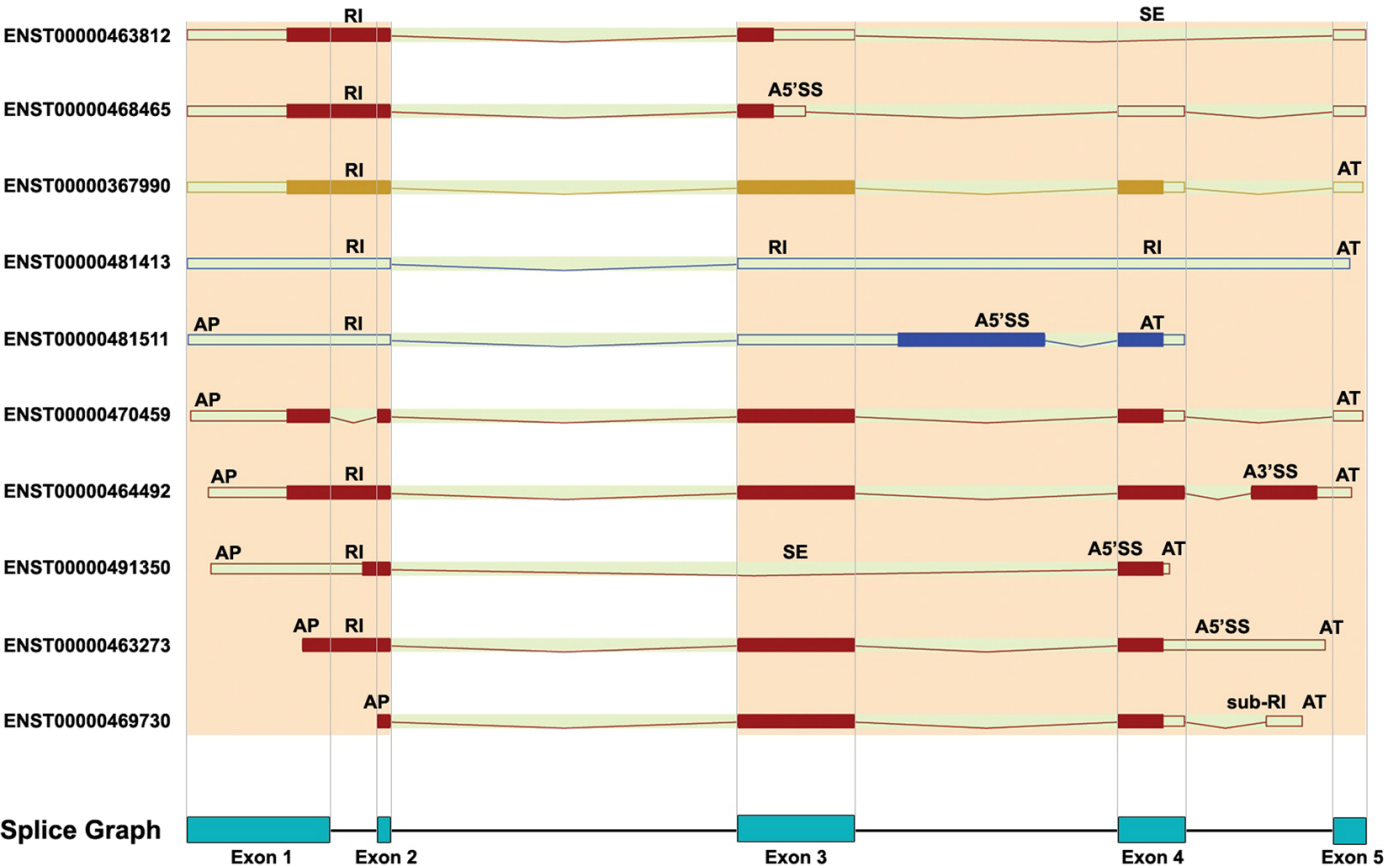
Transcripts of *APOA2* gene and alternative splicing events of each transcript. The main image can be found on http://asia.ensembl.org/Homo_sapiens/Gene/Splice?db=core;g=ENSG00000158874;r=1:161222292-161223631.

This gene contains 10 known transcripts which 8 of them are classified as protein-coding biotypes. We examined the AS occurred types in protein-coding transcripts to evaluate our application performance. The last graph (Fig. 6, SpliceGraph) is formed using our basic rules of SpliceGraph construction. Occurred AS types in transcripts which are extracted based on the arrangement and positioning of exons show the accuracy of our splicing tool results (Table 3). The SpliceGraph includes 5 putative exons. AS types are presented as well as the relevant alternate exons regarding to applied formula in AS detection algorithm of our application.

**Table 3:** Extracted Alternative Splicing events from transcripts of *APOA2* gene. Eight transcripts of all 10 transcripts of *APOA2* gene are from protein-coding biotypes. The first column shows the Ensembl transcript IDs of *APOA2* transcripts and the second column represents AS events occurred on every transcript.

Transcript ID	Splice Event
ENST00000481413	AT, RI(Exon4),RI(Exon3),RI(Exon1),
ENST00000367990	AT,RI(Exon1),
ENST00000463812	SE(Exon4),RI(Exon1),
ENST00000468465	A5′SS(Exon3),RI(Exon1),
ENST00000481511	AP/AT,A5′SS(Exon3),RI(Exon1),
ENST00000470459	AP/AT,
ENST00000464492	AP/AT,A3′SS(Exon5),RI(Exon1),
ENST00000491350	AP/AT,SE(Exon3),A5′SS(Exon4),RI(Exon1),
ENST00000463273	AP/AT,A5′SS(Exon4),RI(Exon1),
ENST00000469730	AP/AT,sub_RI(Exon4),

## Discussion

Alternative splicing of pre-mRNA, as the main cause of the functional diversity in proteins, could also lead to some genetic diseases. Furthermore, AS pattern alteration in samples under treatment has been detected. For instance, exon skipping events are observed after 6TG (6-Thioguanine) treatment throughout the dystrophin transcript^41^. Especially, investigating these alterations in genes with a differential expression which usually appear as transcripts alternation can help to determine the treatments effect on the activity of cells. Sudemycin E which causes a rapid alteration in AS events and consequently changes the overall gene expression and arrests the G2 phase of the cell cycle^42^ is an example of this influence. Regarding the impact of AS events in disease occurrence, efforts to clarify AS events consequences in cellular activity are helpful.

Due to the lack of tools that accept transcript IDs as input for the SpliceGraph building, we decided to compare the criteria for the SpliceGraph formation in some tools regardless of the type and format of the input data. The major part of alternative splicing visualization tools is performing alignment with the reference genome as initial step and then determining the putative exons, based on the criteria of exons expression level, the splice junctions support, genomic coordinate similarity, etc. Regarding the mentioned items, we selected the following tools which are structurally compatible with our application. SpliceGrapher that constructs the SpliceGraph relying on existing gene model annotations. It takes RNA-Seq data as input, and visualizes SpliceGraphs, splice junctions, and read depth. It identifies the splice junction sequence features by spliced-alignment filtering. Vials^43^ is a useful tool that enables researchers to identify abundance of reads associated with exons, recognize splice junctions, and predict isoforms frequency patterns in experimental groups. The tool illustrates the transcripts splicing by the weighted, directed, acyclic graphs modeled using exons genomic coordinates and the splice junctions support (weights). The third selected tool for comparison is SpliceSeq, that is the most similar SpliceGraph design method to our applied method in SpliceDetector application. This software, by summarizing known transcripts in the Ensembl database, constructs a SpliceGraph and then stores them. In the next step, the RNA-seq sample reads are aligned with the pre-deposited reference genome, and genes splice events are extracted using the constructed SpliceGraphs. Our software utilizes transcripts overlapping, a similar method to the SpliceSeq software, and calculates the frequency of splice junctions. However, similar to the three mentioned tools, our application builds SpliceGraphs. In addition to the splice sites support, we used features such as the length of exons and prioritized multiple exons over a continuous exon (including all mentioned multiple exons) with the identical start and end coordinates to improve the SpliceGraph structure, get a better definition of differences between transcripts variants, and recognize all possible exons. Use of this software is as simple as Vials tool which works with the gene names, but we have provided the possibility to enter a set of transcripts in a using process, and we believe it as an advantage for our software. Also, our tool represents a clear view of the alternative splicing events of the query transcript regarding the SpliceGraph and determines the exonic and genomic regions of the events.

We presented the possibility of the investigation of AS patterns in both single and multiple forms: single form for specific transcript investigations and multiple form for cases of having a set of transcripts. Also, an image that represents the query transcript as well as the SpliceGraph constructed from known transcripts of the corresponded gene, gives a clear view of the alternative splicing region and illustrates how the AS events are happened. In addition, in the cases that the Unique transcript reads count of transcripts are input along with transcript IDs, the application provides the possibility to perform a Chi-square Goodness of Fit statistical test to determine significance of alteration rates between Experimental Group and Control Group. The possibility of result exporting in text and Microsoft excel format is considered for results. Methods of application are shown in the practical guide. Data for testing is supplied in the supplemental files (S6–9).

## Conclusion

We developed a practical SpliceGraph-based application for detecting alternative splicing events from transcripts in all model organisms. We eliminated the complicated steps for downloading reference data and using strict command lines arguments in our software to ease extracting AS events straight from transcripts rather than RNA-seq data. Using this software, researchers are able to investigate AS events as the significant factor of alteration in proteins functions through the updated SpliceGraph in each use. The SpliceDetector software is compatible with Windows and needs .NET Framework 4.5. SpliceDetector can be downloaded from https://drive.google.com/open?id=1dlXKzbvxOH3A85_DVR__V2eI5s16-llv or https://www.dropbox.com/s/j5o0og159ig6tej/SpliceDetector%20Executable%20File.rar?dl=0.

## Electronic supplementary material

Supplementary information

Supplementary Dataset S4

Supplementary Dataset S5

Supplementary Dataset S6

S7

S8

Supplementary Dataset S9
