# Supplementary material for: SpliceDetector: a software for detection of alternative splicing events in human and model organisms directly from transcript IDs
Source: Sci Rep. 2018 Mar 22;8:5063. doi: 10.1038/s41598-018-23245-1 (PMC5864913; doi:10.1038/s41598-018-23245-1)
Supplement: Supplementary file 1 — Supplementary information [file 41598_2018_23245_MOESM1_ESM.pdf]

Supplementary Materials

SpliceDetector: a software for detection of alternative splicing events in human and model organisms directly from transcript IDs

Mandana Baharlou Houreh[1], Payam Ghorbani Kalkhajeh[2], Ali Niazi[1], Faezeh Ebrahimi[3], Esmaeil Ebrahimie[1,4,5,6,*]

[1]Institute of Biotechnology, Shiraz University, Shiraz, Iran
[2] Science and Research Branch, Islamic Azad university, Hamedan, Iran
[3]Department of Biology, University of Qom, Qom, Iran
[4]School of Medicine, The University of Adelaide, Adelaide, Australia
[5]School of Information Technology and Mathematical Sciences, Division of Information Technology, Engineering and the Environment, The University of South Australia, Adelaide, SA, Australia
[6]School of Biological Sciences, Faculty of Science and Engineering, Flinders University, Adelaide, SA, Australia
*esmaeil.ebrahimie@adelaide.edu.au

Supplementary Note 1:
List of alternative splicing tools

1. **Transcriptome assemblers**

Row	Tool Name	Description
1	Scripture	a method for transcriptome ab initio reconstruction from RNA-Seq data and an assembled genome
2	Trinity	reconstructs transcripts present in the data and reports alternative splice isoforms and transcripts
3	Trans-ABySS	To map assembled contigs to known transcripts and deduce novel splicing events
4	GRIT	A tool for transcript discovery and quantification via the integrated analysis

2. **Alternative expression**

Row	Tool Name	Description
1	ALEXA-seq	A method for using massively parallel paired-end transcriptome sequencing for 'alternative expression analysis'.
2	MISO	quantifies the expression level of splice variants from RNA-Seq data
3	SplicingCompass	to predict genes that are differentially spliced between two different conditions
4	Flux Capacitor	predicts abundances for transcript molecules and alternative splicing events
5	JuncBASE	to identify and classify alternative splicing events and identifies splice events that are significantly differentially expressed across two or more samples from RNA-Seq data
6	ARH-seq	A discovery tool for differential splicing in case-control studies that is based on the information-theoretic concept of entropy
7	FineSplice	Enhanced splice junction detection and quantification
8	SpliceR	classification of alternative splicing and prediction of coding potential from RNA-Seq data
9	MATS	a computational tool to detect differential alternative splicing events from RNA-Seq data
10	DEXSeq	finding differential exon usage using RNA-seq exon counts between samples with different experimental designs

3. **straight differential expression**

Row	Tool Name	Description
1	edgeR	a package for the analysis of digital gene expression data arising from RNA sequencing technologies
2	DEseq	Determining differentially expressed genes (DEGs) between biological samples
3	htSeq	Assigning aligned reads from an RNA-Seq experiments to exons and genes
4	DEGseq	an R package to identify differentially expressed genes or isoforms for RNA-seq data from different samples

4. based on known splice junctions

Row	Tool Name	Description
1	RUM	an alignment, junction calling, and feature quantification pipeline specifically designed for Illumina RNA-Seq data
2	RNA-MATE	A recursive mapping strategy for high-throughput RNA-sequencing data.
3	PRADA	gene expression levels, quality metrics, detection of unsupervised and supervised fusion transcripts,..
4	OSA	experimental results on Microstructured Optical Fiber (MOF) splicing with a simple method relying on conventional electric-arc splicers
5	MapAL	A tool for RNA-Seq expression profiling
6	IsoformEx	to estimate transcript expression levels and gene expression levels, which takes into account short exons and alternative exons with a weighting scheme
7	Erange	tool for mapping and quantifying Mammalian transcriptomes by RNA-Seq
8	RNASEQR	yields more accurate estimates for gene expression, complete gene structures and new transcript isoforms, as well as more accurate detection of single nucleotide variants
9	SAMMate	a GUI RNA-seq quantification pipeline, allows biomedical researchers to quickly process fasta/fastq and SAM/BAM file
10	SpliceSeq	quantify the inclusion level of each exon and splice junction
11	X-Mate	X-MATE is an updated version of RNA-MATE

5. De novo Splice Aligners

Row	Tool Name	Description
1	ABMapper	De novo Splice Aligners
2	BBMap	De novo Splice Aligners
3	ContextMap	De novo Splice Aligners
4	CRAC	De novo Splice Aligners
5	GSNAP	detect short- and long-distance splicing
6	HISAT	fast and sensitive spliced alignment program for mapping RNA-seq reads
7	HMMSplicer	Tool for Efficient and Sensitive Discovery of Known and Novel Splice Junctions in RNA-Seq Data
8	MapSplice	accurate mapping of RNA-seq reads for splice junction discovery
9	PALMapper	De novo Splice Aligners
10	Pass	De novo Splice Aligners
11	PASSion	De novo Splice Aligners
12	PASTA	De novo Splice Aligners
13	QPALMA	De novo Splice Aligners
14	RAZER	De novo Splice Aligners
15	SeqSaw	De novo Splice Aligners
16	SoapSplice	accurate mapping of RNA-seq reads for splice junction discovery
17	SpliceMap	SpliceMap aligns RNA-seq reads to a reference genome in order to discover splicing junctions
18	SplitSeek	De novo Splice Aligners
19	SuperSplat	De novo Splice Aligners
20	Subread	De novo Splice Aligners
21	Subjunc	De novo Splice Aligners

22	MapNext	**De novo Splice Aligners that also use annotation optionally;** A software tool for both spliced and unspliced alignments of short sequence reads onto reference sequences, and automated SNP detection using neighborhood quality standards
23	OLego	**De novo Splice Aligners that also use annotation optionally;** designed for de novo mapping of spliced mRNA-Seq reads
24	STAR	**De novo Splice Aligners that also use annotation optionally;** Spliced Transcripts Alignment to a Reference (STAR) software by seed clustering and stitching procedure
25	TopHat	**De novo Splice Aligners that also use annotation optionally;** a fast splice junction mapper and aligns RNA-Seq reads using aligner Bowtie
26	G.Mo.R-Se	de novo gene models/ Splice Aligner
27	ASGS	web service facilitating the systematic study of alternatively spliced genes of higher eukaryotes by generating splicing graphs for the compact visual representation of transcript diversity from a single gene

6. General Tools

Row	Software Name	Pipeline & Output
1	Alt Event Finder	skipped exon events
2	Asprofile	determine AS events among multiple samples
3	AStalavista	a platform to investigate the transcriptome diversity across genes, chromosomes, and species
4	CLASS2	assembling transcripts from RNA-seq reads aligned to a genome
5	Cufflinks/Cuffdiff	Finds significant changes in transcript expression, splicing, and promoter use
6	Diceseq	Statistical modeling of isoform splicing dynamics
7	EBChangepoint	change-point model for identifying 3′ and 5′ alternative splicing
8	GESS	for de novo detection of exon-skipping event sites from raw RNA-seq reads
9	LeafCutter	identifies variable intron splicing events from short-read RNA-seq data and finds alternative splicing events
10	LEMONS	Identification of Splice Junctions in Transcriptomes of Organisms Lacking Reference Genomes
11	MAJIQ	• To define splice graphs and known/novel Local Splice Variations (LSV)<br>• Quantifies relative abundance (PSI)
12	RSVP	prediction of alternative isoforms of protein-coding genes
13	SAJR	To analyze splicing and gene expression & To read counts for Genes, Junctions, Segments
14	SplAdder	Identification, quantification and testing of alternative splicing events
15	SpliceJumper	approach for calling splicing junctions
16	SplicePie	analyze non-sequential and multi-step splicing
17	SplicePlot	To visualize alternative splicing and the effects of splicing quantitative trait loci
18	SpliceTrap	quantification of exon inclusion ratios<br>Splice events
19	Splicing Express	• to describe the expression profile of ASEs among all analyzed samples<br>• identifying well-known specific events
20	SUPPA	• Alternative Splicing (AS) events<br>• PSI ("Percentage Spliced In") value for each event

21	SwitchSeq	identification, annotation and visualization of extreme changes in splicing
22	TrueSight	• Splice Junction Detection<br>• sorted alignment results in bam format<br>• inferred splice junctions
23	Vast-tools	profiling and comparing alternative splicing events

7. Other Tools

Row	Tool Name	Description
1	IUTA	to detect genes with differential isoform usages (set of relative abundances of isoforms) between two samples to present a gene's isoform usages between two groups
2	PennSeq	a statistical method that allows each isoform to have its own non-uniform read distribution.
3	FlipFlop	method for de novo transcript discovery and abundance estimation from RNA-Seq data
4	SNPlice	for identifying cis-acting, splice-modulating variants from RNA-seq datasets
5	DiffSplice	tool for discovering and quantitating alternative splicing variants present without relying on annotated transcriptome
6	SigFuge	for identifying genomic loci exhibiting differential transcription patterns across many RNA-seq samples
7	Human Splicing Finder	Splice Site Prediction
8	RegRNA	• Splice Site Prediction<br>• Regulatory RNA Motifs Elements Finder
9	ESEfinder: Exonic Splicing Enhancers finder	• to find Exonic Splicing Enhancer Elements<br>• BranchSites
10	MIT splice predictor	Identification of complete gene structures
11	GeneSplicer	splice site prediction
12	Splice Predictor (DK)	splice site prediction
13	ASPic	• to detect the exon-intron structure of a gene<br>• genomic sequence to the related cluster of ESTs
14	Alternative Splice Site Predictor (ASSP)	Alternative Splicing Prediction
15	SplicePort: An Interactive Splice Site Analysis Tool	splice-site prediction
16	EX-SKIP	• which exonic variant has the highest chance to skip this exon<br>• comparing ESE/ESS profile of a wild-type and a mutated allele
17	RESCUE-ESE	identifying sequences with ESE activity
18	diffsplicing	A method for ranking the genes and transcripts according to the temporal change they show in their expression levels
19	junctionseq	An R package that builds on the statistical techniques used by the well-established DEXSeq package to detect differential usage of both exonic

		regions and splice junctions
20	Non-negative Matrix Factorization Preselection/ NMFP	A preselection method which is designed to improve the accuracy of computational methods in identifying mRNA isoforms
21	jSplice	A junction-centric method that enables de novo extraction of alternative splicing events from RNA-sequencing data
22	sircah	visualizes alternative splicing events using spliced alignments
23	Quantas	A pipeline to analyze alternative splicing using RNA-Seq. gapless and countit are collectively named Quantas.

Supplementary Note 2:
SpliceDetector software source code

```
using System;
using System.Data;
using System.Drawing;
using System.Linq;
using System.Windows.Forms;
using System.Threading;
using System.Diagnostics;
using System.IO;
using System.Data.OleDb;
using System.Xml;
using Accord.Statistics.Testing;
using System.Drawing.Imaging;



namespace TranscriptsSplicingTypes
{
    public partial class Form1 : Form
    {
        public static string PathText = "";
        public static string G_ID = "";
        public static string QueryT_ID = "";
        public static string RefT_ID = "";
        public static DataTable Inputdt = null;
        public static DataTable Inputdt_CT = null;
        public static DataTable Outputdt_CT = null;
        public static DataTable Querydt = null;
        public static DataTable Refdt = null;
        public static DataTable resultdt = null;
        public static DataTable Total_Refdt = null;
        public static DataTable Total_Querydt = null;
        public static DataTable Total_GOdt = null;
        public static string Strand = "";
        public static string WebContent = "";
        public static string Gene_WebContent = "";
        public static string GO_WebContent = "";
        public static string Species_WebContent = "";
        public static string attr = "";
        public static string stableID = "";
        public static string Activity = "";
        static SemaphoreSlim sem1 = new SemaphoreSlim(2);
        static SemaphoreSlim sem2 = new SemaphoreSlim(0);
        static SemaphoreSlim sem3 = new SemaphoreSlim(0);
        static SemaphoreSlim sem4 = new SemaphoreSlim(0);
        static SemaphoreSlim sem5 = new SemaphoreSlim(0);
        static SemaphoreSlim sem6 = new SemaphoreSlim(0);
        static SemaphoreSlim sem7 = new SemaphoreSlim(0);
        static SemaphoreSlim sem8 = new SemaphoreSlim(0);
        static SemaphoreSlim sem9 = new SemaphoreSlim(0);
```

```
int ff0 = 0;
int ff1 = 0;
int ff2 = 0;
int ff3 = 0;

int ErrorVAr = 0;

public Form1()
{
    InitializeComponent();
    this.MouseWheel += new MouseEventHandler(Panel1_MouseWheel);

}
private void Panel1_MouseWheel(object sender, MouseEventArgs e)
{
    panel8.Focus();
    panel8.VerticalScroll.Maximum = 0;
    panel8.AutoScroll = false;
    panel8.HorizontalScroll.Visible = false;
    panel8.AutoScroll = true;
}

public void makeEmpty()
{
    PathText = "";
    G_ID = "";
    QueryT_ID = "";
    RefT_ID = "";
    Querydt = null;
    Refdt = null;
    resultdt = null;
    Strand = "";
    WebContent = "";
    Gene_WebContent = "";
    Species_WebContent = "";
    attr = "";
    stableID = "";

    ff0 = 0;
    ff1 = 0;
    ff2 = 0;
    ff3 = 0;

    ErrorVAr = 0;
}

private void button1_Click(object sender, EventArgs e)
{
    try
    {
        resultdt = makeDataTable(2);
        PathText = openFile();
        var fileNme = Path.GetFileNameWithoutExtension(PathText);
```

```
                OleDbConnection con = this.returnConnection(PathText);
                OleDbCommand oconn = new OleDbCommand("Select * From [" +
"Sheet1" + "$]", con);
                con.Open();

                OleDbDataAdapter sda = new OleDbDataAdapter(oconn);
                Inputdt = new DataTable();
                sda.Fill(Inputdt);

                if ((Activity =="stat") & (Inputdt.Columns.Count < 2))
                {
                    MessageBox.Show("Imported file is not aproperiate");
                    return;
                }
                if ((Activity == "stat") & (Inputdt.Columns.Count > 1))
                {
                    for (int i = 0; i < Inputdt.Rows.Count; i++)
                    {

                        Inputdt.Rows[i][1] =
Math.Round(Convert.ToDouble(Inputdt.Rows[i][1]), 2);

                    }
                }
                dataGridView1.DataSource = Inputdt;
            }
            catch
            {
         MessageBox.Show("Please change file sheet name to 'Sheet1'");
         lB1.Items.Add("Please change file sheet name to 'Sheet1'");
                return;
            }
        }

private string openFile()
{
if (Activity == "GTF")
{
openFileDialog1.DefaultExt = "GTF";
openFileDialog1.Filter = "GTF files (*.gtf)|*.gtf|All files (*.*)|*.*";
}

if (Activity == "GFF3")
{
openFileDialog1.DefaultExt = "GFF3";
openFileDialog1.Filter ="GFF3 files (*.gff3)|*.gff3|All files(*.*)|*.*";}

if ((Activity == "stat") || (Activity == "simple"))
{
openFileDialog1.DefaultExt = "Excel";
openFileDialog1.Filter="Excel files (*.xlsx)|*.xlsx|All files (*.*)|*.*";}
DialogResult result = openFileDialog1.ShowDialog(); // Show the dialog.
if (result == DialogResult.OK) // Test result.
```

```
{
PathText = openFileDialog1.FileName;
                try
                {
                    string text = File.ReadAllText(PathText);
                }
                catch (IOException) {  }
}

return PathText;
        }

public DataTable Make_dt_from_Grid(DataGridView dataGridView1)
{
            DataTable dt = new DataTable();
            if (dataGridView1.Rows.Count > 0)
{
try
{ foreach (DataGridViewColumn col in dataGridView1.Columns)
{
dt.Columns.Add(col.HeaderText, col.ValueType);
}
int count = 0;
foreach (DataGridViewRow row in dataGridView1.Rows)
{
if (count < dataGridView1.Rows.Count - 1)
{
dt.Rows.Add();
foreach (DataGridViewCell cell in row.Cells)
{
dt.Rows[dt.Rows.Count - 1][cell.ColumnIndex] = cell.Value.ToString();}
}
count++; }  }  catch { }
}
return dt;
}

private void webBrowser1_DocumentCompleted(object sender,
WebBrowserDocumentCompletedEventArgs e)
{  try
{
if (webBrowser1.Url.ToString() != "about:blank")
{
filepath = webBrowser1.Document.Body.OuterText.ToString();
}}catch{
ErrorVAr = 1;
lB1.Items.RemoveAt(lB1.Items.Count - 1);
lB1.Items.Insert(lB1.Items.Count, QueryT_ID + ":Without coding protein!");
resultdt.Rows.Add(QueryT_ID, "Without coding protein!");
this.dataGridView3.BeginInvoke((MethodInvoker)delegate () {
dataGridView3.DataSource = resultdt; });
mre.Set();
filepath = null;
return; }   }
```

```
private void webBrowser3_DocumentCompleted(object sender,
WebBrowserDocumentCompletedEventArgs e)
{
try
{
if (webBrowser3.Url.ToString() != "about:blank")
{
WebContent = webBrowser3.Document.Body.OuterText.ToString();  }    }
catch {
ErrorVAr = 2;
lB1.Items.RemoveAt(lB1.Items.Count - 1);
lB1.Items.Insert(lB1.Items.Count, QueryT_ID + ": No coding protein!");

resultdt.Rows.Add(QueryT_ID, "No coding protein!");
this.dataGridView3.BeginInvoke((MethodInvoker)delegate () {
dataGridView3.DataSource = resultdt; });
mre.Set();
WebContent = null;
return;   } }

private DataTable dt_summary(string path, string sheet)
{
DataTable dt = LoadWorksheetInDataTable(@path, sheet);
while (dt.Columns.Count > 4)
{
dt.Columns.RemoveAt(dt.Columns.Count - 1);
}
dt.Rows[0].Delete();
for (int i = 1; i < dt.Rows.Count; i++)
{
if (dt.Rows[i][0].ToString() == "") dt.Rows[i].Delete();
}
dt.AcceptChanges();
return dt;
}

private DataTable LoadWorksheetInDataTable(string fileName, string
sheetName)
{
DataTable sheetData = new DataTable();
using (OleDbConnection conn = this.returnConnection(fileName))
{
conn.Open();
// to retrieve the data using data adapter
OleDbDataAdapter sheetAdapter = new OleDbDataAdapter("select * from [" +
sheetName + "$]", conn);
sheetAdapter.Fill(sheetData);
}
return sheetData;
}
```

```
private OleDbConnection returnConnection(string fileName)
{
string constr = "Provider=Microsoft.ACE.OLEDB.12.0;Data Source=" +
fileName + ";Extended Properties='Excel 12.0 XML;HDR=YES;';";
OleDbConnection con = new OleDbConnection(constr);
return con;
}

public void chang_ExcelFormat(string file)
{
var app = new Microsoft.Office.Interop.Excel.Application();
var wb = app.Workbooks.Open(file);
wb.SaveAs(Filename: file + "x", FileFormat:
Microsoft.Office.Interop.Excel.XlFileFormat.xlOpenXMLWorkbook);
wb.Close();
app.Quit();
}

private void button3_Click(object sender, EventArgs e)
{
string url1 = @"http://asia.ensembl.org/biomart/martservice?query=<?xml
version=" + "\"1.0\"" + " encoding=" + "\"UTF-8\"" + "?><!DOCTYPE Query>";

string url2 = @"<Query  virtualSchemaName =" + "\"default\"" + " formatter
= " + "\"TSV\"" + " header = " + "\"0\"" + " uniqueRows = " + "\"0\"" + "
count = " + "\"\"" + " datasetConfigVersion = " + "\"0.6\"" + "><Dataset
name = " + "\"" + stableID + "_gene_ensembl\"" + " interface = " +
"\"default\"" + "><Filter name = " + "\"ensembl_transcript_id\"" + " value
=\"" + QueryT_ID + "\"/><Attribute name =" + "\"ensembl_gene_id\"" +
"/></Dataset></Query>";

string url3 = url1 + url2;

 webBrowser2.Navigate(url3);
 lB1.Items.RemoveAt(lB1.Items.Count - 1);
 lB1.Items.Insert(lB1.Items.Count, QueryT_ID + ": Retrieving gene ID...");
 }
private void button10_Click(object sender, EventArgs e)
{
try
{
DataTable G_ID_dt = new DataTable();
G_ID_dt = ConvertToDataTable(Gene_WebContent, 1);
G_ID = G_ID_dt.Rows[0][0].ToString();
dataGridView2.DataSource = G_ID_dt;
G_ID_dt.Dispose();
}  catch {  return; }
}

public string onlyOneExon_is_in_range(int G_start, int G_end, int T_start,
int T_end)
{
string result = "";
```

```
if ((T_start == G_start) & (T_end == G_end)) { result = "ExonExist"; }
if ((T_start != G_start) & (T_end == G_end)) { result = "A3'SS(Exon1)"; }
if ((T_start == G_start) & (T_end != G_end)) { result = "A5'SS(Exon1)"; }
return result;
 }


public string new_is_in_range_AP_AT(int T_FirstEnd, int T_LastStart, int
G_FirstEnd, int G_LastStart)
{
            string result = "";
            if ((T_FirstEnd != G_FirstEnd)) { result = "AP"; }
            if ((T_LastStart != G_LastStart)) { result = "AT"; }
            if ((T_FirstEnd != G_FirstEnd) & (T_LastStart != G_LastStart))
{ result = "AP,AT"; }
return result;
}

public string new_is_in_range_AP_AT_Transc(int T_Firststart, int
T_LastEnd, int G_FirstStart, int G_LastEnd)
{            string result = "";
            if ((T_Firststart != G_FirstStart)) { result = "AP"; }
            if ((T_LastEnd != G_LastEnd)) { result = "AT"; }
            if ((T_Firststart != G_FirstStart) & (T_LastEnd != G_LastEnd))
{ result = "AP/AT"; }
return result;
}

public string new_is_in_range(int PG_start, int PG_end, int G_start, int
G_end, int T_start, int T_end, int NG_start, int NG_end)
{
string result = "";
if ((T_start==G_start)&(T_end == G_end)){result="ExonExist";}
if ((T_start>G_end)&(T_end < NG_start)){ result = "sub_RI"; }
if((T_start<=G_end)&(T_start>=G_start)&(T_end<= NG_end)&(T_end>=NG_start))
{ result = "RI"; }

if ((T_start<=PG_end)&(T_start>=PG_start)&(T_end<=G_end)&(T_end>=G_start))
{ result = "ExonExist";}

if ((T_start!=G_start)&(T_end==G_end)&(T_start!=PG_start))
{result="ExonExist"; }

if((T_start!=G_start)&(T_end==G_end)&(T_start!=PG_start)&(T_start>PG_end))
{ result = "A3'SS"; }

if ((T_start == G_start)&(T_end!=G_end)&(T_end!=NG_end)&(T_end<NG_start))
{ result = "A5'SS"; }

if ((T_start<G_end)&(T_start>PG_end)&(T_start!=G_start)&(T_end>G_start)&
(T_end != G_end)) { result = "A5'&3'SS"; }

if ((T_start <= G_start) & (T_end > NG_start)) { result = "RI"; }
```

```
return result;}
public string new_is_in_range_forFirstExon(int G_start, int G_end, int
T_start, int T_end, int NG_start, int NG_end)
{
string result = "";
if ((T_start == G_start) & (T_end == G_end)) { result = "ExonExist"; }
if ((T_start <= G_end) & (T_start >= G_start) & (T_end <= NG_end) & (T_end
>= NG_start)) { result = "RI"; }
if ((T_start== G_end)&(T_end != G_end)&(T_end< NG_start)){result="A5'SS";}
if ((T_start != G_start)&(T_end == G_end)&(T_end < NG_start)){ result =
"ExonExist"; }

return result;
}

public string new_is_in_range_forLastExon(int PG_start, int PG_end, int
G_start, int G_end, int T_start, int T_end)
{
string result = "";

if ((T_start == G_start) & (T_end == G_end)) { result = "ExonExist"; }
if ((T_start != G_start) & (T_end == G_end) & (T_start < PG_end)) { result
= "RI"; }
if ((T_start>PG_end)&(T_start!= G_start)&(T_end>G_start)){result="A3'SS";}
if((T_start>PG_end)&(T_end!=G_end)&(T_start==G_start)){result="ExonExist";
}
if ((T_start > G_end) & (T_end > G_end)) { result = "ExonExist"; }
if ((T_start >= PG_start) & (T_end >= G_end) ) { result = "ExonExist"; }
return result;
}


private void deleteFile(string path)
{
if (File.Exists(@path))
{  File.Delete(@path); }

}
private void button12_Click(object sender, EventArgs e)
{
if (Strand == "R")
{
Querydt = ToReverseDataTable(Querydt);
this.dataGridView4.BeginInvoke((MethodInvoker)delegate () {
dataGridView4.DataSource = Querydt; });
}

string result = "";
string labelresult = "";

DataTable splice_dt = null;
if (Refdt != null)
{
splice_dt = Refdt;
```

```
if (Refdt.Rows.Count == 1)
{ for (int j = 0; j < Querydt.Rows.Count; j++)
{
result = onlyOneExon_is_in_range(
int.Parse(Refdt.Rows[0][2].ToString()),
int.Parse(Refdt.Rows[0][3].ToString()),
int.Parse(Querydt.Rows[j][1].ToString()),
int.Parse(Querydt.Rows[j][2].ToString()));

if ((result != "") & (result != "ExonExist"))
{
labelresult = result + labelresult;
}
}
resultdt.Rows.Add(QueryT_ID, labelresult);
this.dataGridView3.BeginInvoke((MethodInvoker)delegate () {
dataGridView3.DataSource = resultdt; });
resultdt.Columns[0].ColumnName = "Transcript ID";
resultdt.Columns[1].ColumnName = "Splice Event";

lB1.Items.RemoveAt(lB1.Items.Count - 1);
if (ErrorVAr == 0) lB1.Items.Insert(lB1.Items.Count, QueryT_ID + ":
Done;");

 mre.Set();

return;
}  }
            try
            {
                splice_dt.Columns.Add("SpliceType");
            }
            catch
            { return; }

            dataGridView4.DataSource = Querydt;
            dataGridView5.DataSource = Refdt;

            // to collect all Query rows
            foreach (DataRow dr in Refdt.Rows)
            { Total_Refdt.Rows.Add(dr.ItemArray); }

            //to collect all Query rows
            foreach (DataRow dr in Querydt.Rows)
            { Total_Querydt.Rows.Add(dr.ItemArray); }

            for (int j = 0; j < Querydt.Rows.Count; j++)
            {
                for (int z = 0; z < Refdt.Rows.Count; z++)
                {
                    if (z == 0)
                    {
```

```
 result = new_is_in_range_forFirstExon(

int.Parse(Refdt.Rows[z][2].ToString()),
int.Parse(Refdt.Rows[z][3].ToString()),
int.Parse(Querydt.Rows[j][1].ToString()),
int.Parse(Querydt.Rows[j][2].ToString()),
int.Parse(Refdt.Rows[z + 1][2].ToString()),
int.Parse(Refdt.Rows[z + 1][3].ToString()));
                    }
                    else
                    {
                        if (z == Refdt.Rows.Count - 1)

                        {
                            result = new_is_in_range_forLastExon(
                            int.Parse(Refdt.Rows[z - 1][2].ToString()),
                            int.Parse(Refdt.Rows[z - 1][3].ToString()),

int.Parse(Refdt.Rows[z][2].ToString()),
int.Parse(Refdt.Rows[z][3].ToString()),
int.Parse(Querydt.Rows[j][1].ToString()),
int.Parse(Querydt.Rows[j][2].ToString())
); }
                        else
                        {
                            result = new_is_in_range(
                            int.Parse(Refdt.Rows[z - 1][2].ToString()),
                            int.Parse(Refdt.Rows[z - 1][3].ToString()),

int.Parse(Refdt.Rows[z][2].ToString()),
int.Parse(Refdt.Rows[z][3].ToString()),
int.Parse(Querydt.Rows[j][1].ToString()),
int.Parse(Querydt.Rows[j][2].ToString()),
int.Parse(Refdt.Rows[z + 1][2].ToString()),
int.Parse(Refdt.Rows[z + 1][3].ToString()));
}
}
if (result != "")
{
 if ((result == "ExonExist"))
{
splice_dt.Rows[z][4] = result + "(" + splice_dt.Rows[z][1].ToString()+")";
}
 else if ((result == "sub_RI"))
{
if (splice_dt.Rows[z][4].ToString().Contains("ExonExist"))
{
splice_dt.Rows[z][4]= "sub_RI"+"("+splice_dt.Rows[z][1].ToString()+")";}
else
{
splice_dt.Rows[z][4]="SE/sub_RI"+"(" +
splice_dt.Rows[z][1].ToString()+")";
}
```

```
result = splice_dt.Rows[z][4].ToString();
labelresult = result + "," + labelresult;
}
else
{
splice_dt.Rows[z][4] = ",";
labelresult = result + "(" + splice_dt.Rows[z][1].ToString() + ")" + "," +
labelresult;  }

 }     }     }

for (int k = 0; k < splice_dt.Rows.Count; k++)
{
if ((int.Parse(Refdt.Rows[k][3].ToString().Replace(",", "")) >
int.Parse(Querydt.Rows[0][1].ToString().Replace(",", ""))) &
                    (int.Parse(Refdt.Rows[k][3].ToString().Replace(",",
"")) <= int.Parse(Querydt.Rows[Querydt.Rows.Count -
1][2].ToString().Replace(",", ""))))
{
if (splice_dt.Rows[k][4].ToString() == "")
{
splice_dt.Rows[k][4] = "SE" + "(" + splice_dt.Rows[k][1].ToString() + ")";
labelresult = splice_dt.Rows[k][4] + "," + labelresult;
}                }            }

result =
new_is_in_range_AP_AT_Transc(int.Parse(Querydt.Rows[0][1].ToString()),
int.Parse(Querydt.Rows[Querydt.Rows.Count - 1][2].ToString()),
int.Parse(Refdt.Rows[0][2].ToString()),
int.Parse(Refdt.Rows[Refdt.Rows.Count - 1][3].ToString()));

            if (result != "")
            {
splice_dt.Rows[0][4] = splice_dt.Rows[0][4].ToString() + "," + result;
labelresult = result + "," + labelresult;

            }

splice_dt.Dispose();

resultdt.Columns[0].ColumnName = "Transcript ID";
resultdt.Columns[1].ColumnName = "Splice Event";

if (ErrorVAr == 0)
{
if (labelresult != "")
resultdt.Rows.Add(QueryT_ID, labelresult);
else     resultdt.Rows.Add(QueryT_ID, "No Splice");
}
else{resultdt.Rows.Add(QueryT_ID, "NA");}

ErrorVAr = 0;
```

```
this.dataGridView3.BeginInvoke((MethodInvoker)delegate () {
dataGridView3.DataSource = resultdt; });
mre.Set();
}

private void Form1_Load(object sender, EventArgs e)
{
toolBox1.Left = 6000;
System.Windows.Forms.ToolTip ToolTip1 = new
System.Windows.Forms.ToolTip();
ToolTip1.SetToolTip(label38, "goodness of fit");

System.Windows.Forms.ToolTip ToolTip2 = new
System.Windows.Forms.ToolTip();
ToolTip2.SetToolTip(label2, "Exon Skipping");

System.Windows.Forms.ToolTip ToolTip3 = new
System.Windows.Forms.ToolTip();
ToolTip3.SetToolTip(label9, "Intron Retention");

System.Windows.Forms.ToolTip ToolTip4 = new
System.Windows.Forms.ToolTip();
ToolTip4.SetToolTip(label10, "Alternative 3' splice site");


System.Windows.Forms.ToolTip ToolTip5 = new
System.Windows.Forms.ToolTip();
ToolTip5.SetToolTip(label11, "Alternative 5' splice site");


System.Windows.Forms.ToolTip ToolTip6 = new
System.Windows.Forms.ToolTip();
ToolTip6.SetToolTip(label12, "Sub-Intron Retention");

System.Windows.Forms.ToolTip ToolTip7 = new
System.Windows.Forms.ToolTip();
ToolTip7.SetToolTip(label13, "Alternative Promoters");

System.Windows.Forms.ToolTip ToolTip8 = new
System.Windows.Forms.ToolTip();
ToolTip8.SetToolTip(label14, "Alternative Terminators");

pictureBox2.Height = panel8.Height-10;
pictureBox2.Width = panel8.Width-10;
groupBox9.Height = groupBox12.Height;
groupBox9.Width = groupBox12.Width;
groupBox9.Left = groupBox12.Left;
groupBox9.Top = groupBox12.Top;
}
private void button15_Click(object sender, EventArgs e)
{

string url1 = @"http://asia.ensembl.org/biomart/martservice?query=<?xml
version=" + "\"1.0\"" + " encoding=" + "\"UTF-8\"" + "?><!DOCTYPE Query>";
```

```
string url2 = @"<Query  virtualSchemaName =" + "\"default\"" + " formatter
= " + "\"TSV\"" + " header = " + "\"0\"" + " uniqueRows = " + "\"0\"" + "
count = " + "\"\"" + " datasetConfigVersion = " + "\"0.6\"" + "><Dataset
name = " + "\"" + stableID + "_gene_ensembl\"" + " interface = " +
"\"default\"" + "><Filter name = " + "\"transcript_biotype\"" + " value
=\"protein_coding\"" + "/><Filter name = " + "\"ensembl_gene_id\"" + "
value =\"" + G_ID + "\"/><Attribute name =" + "\"ensembl_exon_id\"" +
"/><Attribute name =" + "\"exon_chrom_start\"" + "/><Attribute name =" +
"\"exon_chrom_end\"" + "/><Attribute name =" + "\"strand\"" +
"/><Attribute name =" + "\"rank\"" + "/></Dataset></Query>";

string url3 = url1 + url2;
webBrowser1.Navigate(url3);

}


public DataTable makeDataTable(int numberOfColumns)
{
DataTable tbl = new DataTable();
for (int col = 0; col < numberOfColumns; col++)
tbl.Columns.Add(new DataColumn("Column" + (col + 1).ToString()));
return tbl;
 }

public DataTable ConvertToDataTable(string filePath, int numberOfColumns)
{
try
{
DataTable tbl = new DataTable();

for (int col = 0; col < numberOfColumns; col++)
tbl.Columns.Add(new DataColumn("Column" + (col + 1).ToString()));
string[] lines = filePath.Split(new string[] { "\r\n", "\n" },
StringSplitOptions.None);

foreach (string line in lines)
{
                    var cols = line.Split('\t');
                    DataRow dr = tbl.NewRow();
                for (int cIndex = 0; cIndex < numberOfColumns; cIndex++)
                    {
                    dr[cIndex] = cols[cIndex];
                    }
                    tbl.Rows.Add(dr);
                 }

              return tbl;
             }
             catch
             {
              lB1.Items.Add("Ensembl Transcript ID not found!");
              return null;
```

```
            }
        }

public DataTable ToReverseDataTable(DataTable dt)
{
DataTable Rdt = new DataTable();
for (int col = 0; col < dt.Columns.Count; col++)
Rdt.Columns.Add(new DataColumn("Column" + (col + 1).ToString()));

for (int row = dt.Rows.Count - 1; row > -1; row--)
{
DataRow dr = Rdt.NewRow();

dr[0] = dt.Rows[row][0].ToString();
dr[1] = dt.Rows[row][1].ToString();
dr[2] = dt.Rows[row][2].ToString();
dr[3] = dt.Rows[row][3].ToString();
Rdt.Rows.Add(dr);
}
return Rdt;
}

string filepath;
string Tfilepath;
private void button16_Click(object sender, EventArgs e)
{
            DataTable Exondt = new DataTable();
            try
            {
                Exondt = ConvertToDataTable(filepath, 5);
                if (Exondt.Rows[0][3].ToString() == "1")
                { Strand = "F"; }
                else   { Strand = "R"; }            }
            catch
            {
                if (ErrorVAr == 0)
                {
                    resultdt.Rows.Add(QueryT_ID, "NA");
                    resultdt.Columns[0].ColumnName = "Transcript ID";
                    resultdt.Columns[1].ColumnName = "Splice Event";
                    this.dataGridView3.BeginInvoke((MethodInvoker)delegate
() { dataGridView3.DataSource = resultdt; });
                    lB1.Items.RemoveAt(lB1.Items.Count - 1);
                    lB1.Items.Insert(lB1.Items.Count, QueryT_ID + ":
NA;");
mre.Set();}
return; }
int N = 0;
            for (int y = 0; y < Exondt.Rows.Count; y++)
            {
                if (Exondt.Rows[y][4].ToString() == "1") N++;
            }
            if (N == 1)
            {
```

```
lB1.Items.RemoveAt(lB1.Items.Count - 1);
lB1.Items.Insert(lB1.Items.Count, QueryT_ID + ": only one coding protein transcript!");                resultdt.Rows.Add(QueryT_ID, "only one coding protein transcript");
this.dataGridView3.BeginInvoke((MethodInvoker)delegate () { dataGridView3.DataSource = resultdt; });
mre.Set();
return;
}
DataTable dt = new DataTable();
for (int col = 0; col < 3; col++)
dt.Columns.Add(new DataColumn("Column" + (col + 1).ToString()));
DataRow dr = dt.NewRow();
for (int i = 0; i < Exondt.Rows.Count; i++)
{
                dr = dt.NewRow();
                dr[0] = Exondt.Rows[i][1].ToString();
                dr[1] = "Start";
                dr[2] = Exondt.Rows[i][0].ToString();
                dt.Rows.Add(dr);
}
for (int i = 0; i < Exondt.Rows.Count; i++)
{
                dr = dt.NewRow();
                dr[0] = Exondt.Rows[i][2].ToString();
                dr[1] = "End";
                dr[2] = Exondt.Rows[i][0].ToString();
                dt.Rows.Add(dr);
}
            DataView dv = dt.DefaultView;
            dv.Sort = dv.Table.Columns[0].ColumnName;
            DataTable sortedDT = dv.ToTable();

            DataView dv1 = new DataView(sortedDT);
            DataTable dtGroup = dv1.ToTable(false, new string[] { "Column1", "Column2", "Column3" });
            dtGroup.Columns.Add("Count", typeof(int));

looping thru distinct values for the group, counting
foreach (DataRow dr1 in dtGroup.Rows)
{
dr1["Count"] = dtGroup.Compute("Count(" + "Column1" + ")", "Column1" + " = '" + dr1["Column1"] + "'");
}

dtGroup = dtGroup.AsEnumerable().GroupBy(r => new { Col1 = r["Column1"] }).Select(g => g.First()).CopyToDataTable();

dataGridView6.DataSource = dtGroup;

            DataTable Graphdt = new DataTable();
            Graphdt.Columns.Add("c1");
            Graphdt.Columns.Add("c2");
            Graphdt.Columns.Add("c3");
```

```
            Graphdt.Columns.Add("c4");
            DataTable Tempdt = new DataTable();
            Tempdt.Columns.Add("c1");
            Tempdt.Columns.Add("c2");
            Tempdt.Columns.Add("c3");
            Tempdt.Columns.Add("c4");
            string currentStartEndLabel = "";
            string secondStartEndLabel = "";
            float currentCPLabel = 0;
            float secondCPLabel = 0;

            //correction

for (int k = 0; k < dtGroup.Rows.Count - 1; k++)
{
if (k == dtGroup.Rows.Count - 2)
{
if (Graphdt.Rows[Graphdt.Rows.Count - 1][1].ToString() == "Start")
{
DataRow G_dr = Graphdt.NewRow();
G_dr[0] = dtGroup.Rows[dtGroup.Rows.Count - 1][0].ToString();
G_dr[1] = dtGroup.Rows[dtGroup.Rows.Count - 1][1].ToString();
G_dr[2] = dtGroup.Rows[dtGroup.Rows.Count - 1][2].ToString();
G_dr[3] = dtGroup.Rows[dtGroup.Rows.Count - 1][3].ToString();
Graphdt.Rows.Add(G_dr);
}
}

                currentStartEndLabel = dtGroup.Rows[k][1].ToString();
                secondStartEndLabel = dtGroup.Rows[k + 1][1].ToString();
                currentCPLabel =
float.Parse(dtGroup.Rows[k][3].ToString());
secondCPLabel = float.Parse(dtGroup.Rows[k + 1][3].ToString());
DataRow tempdr;
if ((currentStartEndLabel == "Start") & (secondStartEndLabel == "End"))
{
if (currentStartEndLabel == "Start")
{
                        tempdr = Tempdt.NewRow();
                        tempdr[0] = dtGroup.Rows[k][0].ToString();
                        tempdr[1] = dtGroup.Rows[k][1].ToString();
                        tempdr[2] = dtGroup.Rows[k][2].ToString();
                        tempdr[3] = dtGroup.Rows[k][3].ToString();
                        Tempdt.Rows.Add(tempdr);
                        Tempdt.DefaultView.Sort = "c1 ASC";
                        dataGridView2.DataSource = Tempdt;
                        var maxRow = Tempdt.Select("c4 = Max(c4)");
                        DataRow G_dr = Graphdt.NewRow();
                        G_dr[0] = maxRow[0][0].ToString();
                        G_dr[1] = maxRow[0][1].ToString();
                        G_dr[2] = maxRow[0][2].ToString();
                        G_dr[3] = maxRow[0][3].ToString();
                        Graphdt.Rows.Add(G_dr);
                        Tempdt.Clear();
```

```
                    }

                }
                else
                {
                    if (currentStartEndLabel == "Start")
                    {
                        tempdr = Tempdt.NewRow();
                        tempdr[0] = dtGroup.Rows[k][0].ToString();
                        tempdr[1] = dtGroup.Rows[k][1].ToString();
                        tempdr[2] = dtGroup.Rows[k][2].ToString();
                        tempdr[3] = dtGroup.Rows[k][3].ToString();
                        Tempdt.Rows.Add(tempdr);
                    }
                }
            }

            for (int k = 0; k < dtGroup.Rows.Count - 1; k++)
            {
                currentStartEndLabel = dtGroup.Rows[k][1].ToString();
                secondStartEndLabel = dtGroup.Rows[k + 1][1].ToString();
                currentCPLabel =
float.Parse(dtGroup.Rows[k][3].ToString());
secondCPLabel = float.Parse(dtGroup.Rows[k + 1][3].ToString());
DataRow tempdr;
if ((currentStartEndLabel == "End") & (secondStartEndLabel == "Start"))
{
if (currentStartEndLabel == "End")
{                       tempdr = Tempdt.NewRow();
                        tempdr[0] = dtGroup.Rows[k][0].ToString();
                        tempdr[1] = dtGroup.Rows[k][1].ToString();
                        tempdr[2] = dtGroup.Rows[k][2].ToString();
                        tempdr[3] = dtGroup.Rows[k][3].ToString();
                        Tempdt.Rows.Add(tempdr);
                        Tempdt.DefaultView.Sort = "c1 DESC";
                        var maxRow = Tempdt.Select("c4 = Max(c4));
                        DataRow G_dr = Graphdt.NewRow();
                        G_dr[0] = maxRow[0][0].ToString();
                        G_dr[1] = maxRow[0][1].ToString();
                        G_dr[2] = maxRow[0][2].ToString();
                        G_dr[3] = maxRow[0][3].ToString();
                        Graphdt.Rows.Add(G_dr);
                        Tempdt.Clear();
                    }
                }
                else
                {
                    if (currentStartEndLabel == "End")
                    {
                        tempdr = Tempdt.NewRow();
                        tempdr[0] = dtGroup.Rows[k][0].ToString();
                        tempdr[1] = dtGroup.Rows[k][1].ToString();
                        tempdr[2] = dtGroup.Rows[k][2].ToString();
                        tempdr[3] = dtGroup.Rows[k][3].ToString();
```

```
                        Tempdt.Rows.Add(tempdr);
                    }
                }
            }
            DataView Gdv = Graphdt.DefaultView;
            Gdv.Sort = Gdv.Table.Columns[0].ColumnName;
            DataTable G_sortedDT = Gdv.ToTable();
            dataGridView6.DataSource = G_sortedDT;
            int c = 0;

            //to delete exons with only one repeat
            while (c < G_sortedDT.Rows.Count - 1)
            {
                for (int k = 0; k < G_sortedDT.Rows.Count - 1; k++)
                {
currentStartEndLabel = G_sortedDT.Rows[k][1].ToString();
secondStartEndLabel = G_sortedDT.Rows[k + 1][1].ToString();
currentCPLabel = int.Parse(G_sortedDT.Rows[k][3].ToString());
secondCPLabel = int.Parse(G_sortedDT.Rows[k + 1][3].ToString());

if ((secondStartEndLabel == "End") & (currentStartEndLabel == "Start") &
(currentCPLabel == 1) & (secondCPLabel == 1))
                    {   G_sortedDT.Rows[k].Delete();
                        G_sortedDT.AcceptChanges();
                        G_sortedDT.Rows[k].Delete();
                        G_sortedDT.AcceptChanges();
                    }
                } c++;
            }

           DataTable Graph_Refdt = new DataTable();
           Graph_Refdt.Columns.Add("Row");
           Graph_Refdt.Columns.Add("ExonID");
           Graph_Refdt.Columns.Add("Start");
           Graph_Refdt.Columns.Add("End");
           int row = 1;
           int cg=0 ;
           if (G_sortedDT.Rows[0][1].ToString() == "End") cg = 1;
           for (int g = cg;  g < G_sortedDT.Rows.Count; g++)
           {
                DataRow G_dr = Graph_Refdt.NewRow();
                G_dr[0] = QueryT_ID;
                G_dr[1] = "Exon" + row++;
                G_dr[2] = G_sortedDT.Rows[g][0].ToString();
                G_dr[3] = G_sortedDT.Rows[g + 1][0].ToString();
                Graph_Refdt.Rows.Add(G_dr);
                g++;
            }     Refdt = Graph_Refdt;        }

public void ExportToExcel(DataTable dt, string path)
{
try
{
// Bind table data to Stream Writer to export data to respective folder
```

```
StreamWriter wr = new StreamWriter(path + @"\SpliceResults.xls");
// Write Columns to excel file
for (int i = 0; i < dt.Columns.Count; i++)
{
wr.Write(dt.Columns[i].ToString().ToUpper() + "\t");
}
wr.WriteLine();
//write rows to excel file
for (int i = 0; i < (dt.Rows.Count); i++)
{
for (int j = 0; j < dt.Columns.Count; j++)
{
if (dt.Rows[i][j] != null)
{
wr.Write(Convert.ToString(dt.Rows[i][j]) + "\t");
                        }
                        else
                        {    wr.Write("\t");  }
                    }
                    wr.WriteLine();
                }
                wr.Close();

MessageBox.Show(@"Data Exported Successfully as 'c:\SpliceResults.xls'");
lB1.Items.Add(@"Data Exported Successfully as 'c:\SpliceResults.xls'");
}
catch (Exception ex) { throw ex; }
}
        int i = 0;
        ManualResetEvent mre = new ManualResetEvent(false);
        private void button4_Click_1(object sender, EventArgs e)
        {
            resultdt = makeDataTable(2);

            Total_Refdt = new DataTable();
            Total_Refdt.Columns.Add("Row");
            Total_Refdt.Columns.Add("ExonID");
            Total_Refdt.Columns.Add("Start");
            Total_Refdt.Columns.Add("End");
            Total_Refdt.Columns.Add("SpliceEvents");
            Total_Querydt = new DataTable();
            Total_Querydt.Columns.Add("Column1");
            Total_Querydt.Columns.Add("Column2");
            Total_Querydt.Columns.Add("Column3");
            Total_Querydt.Columns.Add("Column4");
            Total_GOdt = new DataTable();
            Total_GOdt.Columns.Add("TranscriptID");
            Total_GOdt.Columns.Add("GeneName");
            Total_GOdt.Columns.Add("GO");

            lB1.Items.Clear();
            if (rB1.Checked == true)
            {
              makeEmpty();
```

```
                resultdt = makeDataTable(2);
                pB1.Maximum = 1;
                pB1.Value = 0;
                QueryT_ID = textBox2.Text;
                 //checking transcript format
if ((QueryT_ID.Substring(0, 3) != "ENS") || (QueryT_ID.Length < 15))
                 {  ErrorVAr = 1;  }
lB1.Items.Insert(0, QueryT_ID + ": Retrieving gene ID...");
button20_Click(null, null);
this.statusStrip1.BeginInvoke((MethodInvoker)delegate(){pB1.Value = 1; });
                 ff0 = 0;
                 ff1 = 0;
                 ff2 = 0;
                 ff3 = 0;
}
            if (rB2.Checked == true)
            {
                if (dataGridView1.RowCount == 0)
                {
                    MessageBox.Show("Please import excel file");
                    lB1.Items.Add("Please import excel file");
                    return;

                }
                pB1.Maximum = Inputdt.Rows.Count;
                pB1.Value = 0;
                ErrorVAr = 0;
Thread thread1 = new Thread(new ThreadStart(  () =>
{  while (i < Inputdt.Rows.Count)
{
mre.Reset();
QueryT_ID = Inputdt.Rows[i][0].ToString();
//checking transcript format
if ((QueryT_ID.Substring(0, 3) != "ENS") || (QueryT_ID.Length < 15))
{MessageBox.Show("Please enter correct form of Transcript ID");
ErrorVAr = 1;}
this.lB1.BeginInvoke((MethodInvoker)delegate () {
lB1.Items.Insert(lB1.Items.Count,QueryT_ID+": Retrieving gene ID..."); });
button20_Click(null, null);
this.statusStrip1.BeginInvoke((MethodInvoker)delegate () { pB1.Value = i;
});
ff0 = 0;
ff1 = 0;
ff2 = 0;
ff3 = 0;
++i;
mre.WaitOne();
}
MessageBox.Show("Done!");
if (Activity == "stat")
{
this.StatBox.BeginInvoke((MethodInvoker)delegate () { StatBox.Visible =
true; });
}
```

```
}
));

thread1.Start();}

}
private void button6_Click_1(object sender, EventArgs e)
{
string url1 = @"http://asia.ensembl.org/biomart/martservice?query=<?xml
version=" + "\"1.0\"" + " encoding=" + "\"UTF-8\"" + "?><!DOCTYPE Query>";

string url2 = @"<Query  virtualSchemaName =" + "\"default\"" + " formatter
= " + "\"TSV\"" + " header = " + "\"0\"" + " uniqueRows = " + "\"0\"" + "
count = " + "\"\"" + " datasetConfigVersion = " + "\"0.6\"" + "><Dataset
name = " + "\"" + stableID + "_gene_ensembl\"" + " interface = " +
"\"default\"" + "><Filter name = " + "\"ensembl_transcript_id\"" + " value
=\"" + QueryT_ID + "\"/><Attribute name =" + "\"ensembl_transcript_id\"" +
"/><Attribute name =" + "\"exon_chrom_start\"" + "/><Attribute name =" +
"\"exon_chrom_end\"" + "/><Attribute name =" + "\"strand\"" +
"/></Dataset></Query>";
string url3 = url1 + url2;
webBrowser3.Navigate(url3);

}

 private void button7_Click_1(object sender, EventArgs e)
{ try
            {   Tfilepath = WebContent;
                Querydt = ConvertToDataTable(Tfilepath, 4);
                if (Querydt.Rows[0][3].ToString() == "1")
                {
                    Strand = "F";
                    DataView Qdv = Querydt.DefaultView;
                    Qdv.Sort = Qdv.Table.Columns[1].ColumnName + "  ASC";
                    Querydt = Qdv.ToTable();
                }
                else
                {
                    Strand = "R";
                    DataView Qdv = Querydt.DefaultView;
                    Qdv.Sort = Qdv.Table.Columns[1].ColumnName + "  DESC";
                    Querydt = Qdv.ToTable();
                }
           }
            catch  {    return;    }
        }
 private void button20_Click(object sender, EventArgs e)
 {
button5_Click(null, null);
s0();
}
void s0()
{
webBrowser4.DocumentCompleted += (sender, e) =>
```

```
{
                if (ff0 == 0)
                {
                    button3_Click(null, null);
                    s1();
                    ff0 = 1;
                }
 };

}
void s1()
{            webBrowser2.DocumentCompleted += (sender, e) =>
            {
                if (ff1 == 0)
                {
                    button10_Click(null, null);
                    button6_Click_1(null, null);
if (ErrorVAr == 0)
{
lB1.Items.RemoveAt(lB1.Items.Count - 1);
lB1.Items.Insert(lB1.Items.Count,QueryT_ID+":Retrieving transcript
exons...");  }
s2();
ff1 = 1;
}   };
}

void s2()
{
webBrowser3.DocumentCompleted += (sender, e) =>
{                if (ff2 == 0)
                {
                    button7_Click_1(null, null);
                    button15_Click(null, null);
                    if (ErrorVAr == 0)
                    {
lB1.Items.RemoveAt(lB1.Items.Count - 1);
lB1.Items.Insert(lB1.Items.Count, QueryT_ID +": Retrieving all exons...");
}
s3();
ff2 = 1;
}
};
}


void s3()
{
webBrowser1.DocumentCompleted += (sender, e) =>
{
if (ff3 == 0)
{
if (ErrorVAr == 0)
{
```

```
lB1.Items.RemoveAt(lB1.Items.Count - 1);
lB1.Items.Insert(lB1.Items.Count, QueryT_ID + ": Splice graph
construction...");
}

                    button16_Click(null, null);
                    button12_Click(null, null);
lB1.Items.RemoveAt(lB1.Items.Count - 1);
lB1.Items.Insert(lB1.Items.Count, QueryT_ID + ": Done;");
                    ff3 = 1;
                }            };        }



private void button11_Click(object sender, EventArgs e)
{
ExportToExcel(resultdt, @"C:\");
}

private void spliceTypesToolStripMenuItem_Click(object sender, EventArgs
e)
{
Process.Start(@"Splice_Types.pdf");
}

private void button2_Click(object sender, EventArgs e)
{
WriteToTextfile(resultdt, @"c:\SpliceResults.txt");
}

public static void WriteToTextfile(DataTable dt, string filePath)
{
            int i = 0;
            StreamWriter swExtLogFile = new StreamWriter(filePath, true);
            swExtLogFile.Write(Environment.NewLine);
            foreach (DataRow row in dt.Rows)
            {
                object[] array = row.ItemArray;
                for (i = 0; i < array.Length - 1; i++)
                {
                    swExtLogFile.Write(array[i].ToString() + ";");
                }
                swExtLogFile.Write(array[i].ToString());
                swExtLogFile.WriteLine();

}
swExtLogFile.Write("*****END OF DATA****" + DateTime.Now.ToString());
swExtLogFile.Flush();
swExtLogFile.Close();
MessageBox.Show(@"Data Exported Successfully as 'c:\SpliceResults.txt'");

}
```

```
private void webBrowser2_DocumentCompleted(object sender,
WebBrowserDocumentCompletedEventArgs e)
{
try
{
if (webBrowser2.Url.ToString() != "about:blank")
{
Gene_WebContent = webBrowser2.Document.Body.OuterText.ToString();
}    }
catch
{
lB1.Items.Add("Please enter correct form of Transcript ID");
resultdt.Rows.Add(QueryT_ID, "NA");
return;
}
}

private void exitToolStripMenuItem_Click(object sender, EventArgs e)
{
Form1.ActiveForm.Close();
}

private void webBrowser4_DocumentCompleted(object sender,
WebBrowserDocumentCompletedEventArgs e)
{
try
{
if (webBrowser4.Url.ToString() != "about:blank")
{
Species_WebContent = webBrowser4.Document.Body.OuterText.ToString();
                    XmlDocument doc = new XmlDocument();
                    string attr = "";
                    doc.LoadXml("<xml>" + Species_WebContent + "</xml>");
XmlNodeList elemList = doc.GetElementsByTagName("data");
                    for (int i = 0; i < elemList.Count; i++)
                    {
                        attr = elemList[i].Attributes["species"].Value;

                    }
int pos = int.Parse(attr.IndexOf("_").ToString());
stableID = attr.Substring(0, 1) + attr.Substring(pos + 1);
}

}  catch   {   return;  }
}


private void button5_Click(object sender, EventArgs e)
{
string url1 = @"http://rest.ensembl.org/lookup/id/" + QueryT_ID +
"/BRCA2?content-type=text/xml;format=condensed;db_type=core";
webBrowser4.Navigate(url1);
}
```

```
private void button14_Click(object sender, EventArgs e)
{
            resultdt.Columns.Add(new DataColumn("ControlReadCount"));
            resultdt.Columns.Add(new DataColumn("TreatmentReadCount"));
            resultdt.Columns.Add(new DataColumn("Control_SE"));
            resultdt.Columns.Add(new DataColumn("Control_RI"));
            resultdt.Columns.Add(new DataColumn("Control_A3"));
            resultdt.Columns.Add(new DataColumn("Control_A5"));
            resultdt.Columns.Add(new DataColumn("Control_sub"));
            resultdt.Columns.Add(new DataColumn("Control_AP"));
            resultdt.Columns.Add(new DataColumn("Control_AT"));

            resultdt.Columns.Add(new DataColumn("Treatment_SE"));
            resultdt.Columns.Add(new DataColumn("Treatment_RI"));
            resultdt.Columns.Add(new DataColumn("Treatment_A3"));
            resultdt.Columns.Add(new DataColumn("Treatment_A5"));
            resultdt.Columns.Add(new DataColumn("Treatment_sub"));
            resultdt.Columns.Add(new DataColumn("Treatment_AP"));
            resultdt.Columns.Add(new DataColumn("Treatment_AT"));


for (int j = 0; j < resultdt.Rows.Count; j++)
{
for (int z = 0; z < resultdt.Rows.Count; z++)
{
if (Inputdt.Rows[j][0].ToString() == resultdt.Rows[z][0].ToString())
{
resultdt.Rows[z][2] =
Math.Ceiling(float.Parse(Inputdt.Rows[j][1].ToString()));
resultdt.Rows[z][3] =
Math.Ceiling(float.Parse(Inputdt.Rows[j][2].ToString()));
}}}
}

private void button17_Click(object sender, EventArgs e)
        {
            int SE_counter = 0;
            int RI_counter = 0;
            int A3_counter = 0;
            int A5_counter = 0;
            int sub_counter = 0;
            int AP_counter = 0;
            int AT_counter = 0;

            int SE_T_counter = 0;
            int RI_T_counter = 0;
            int A3_T_counter = 0;
            int A5_T_counter = 0;
            int sub_T_counter = 0;
            int AP_T_counter = 0;
            int AT_T_counter = 0;

            int SE_C_counter = 0;
```

```
            int RI_C_counter = 0;
            int A3_C_counter = 0;
            int A5_C_counter = 0;
            int sub_C_counter = 0;
            int AP_C_counter = 0;
            int AT_C_counter = 0;


for (int i = 0; i < resultdt.Rows.Count; i++)
{
SE_counter = countChars(resultdt.Rows[i][1].ToString(), "SE");
RI_counter = countChars(resultdt.Rows[i][1].ToString(), ",RI");
A3_counter = countChars(resultdt.Rows[i][1].ToString(), "A3");
A5_counter = countChars(resultdt.Rows[i][1].ToString(), "A5");
sub_counter = countChars(resultdt.Rows[i][1].ToString(), "sub_RI");
AP_counter = countChars(resultdt.Rows[i][1].ToString(), "AP");
AT_counter = countChars(resultdt.Rows[i][1].ToString(), "AT");

resultdt.Rows[i][4] = (SE_counter *
int.Parse(resultdt.Rows[i][2].ToString()));
resultdt.Rows[i][5] = (RI_counter *
int.Parse(resultdt.Rows[i][2].ToString()));
resultdt.Rows[i][6] = (A3_counter *
int.Parse(resultdt.Rows[i][2].ToString()));
resultdt.Rows[i][7] = (A5_counter *
int.Parse(resultdt.Rows[i][2].ToString()));
resultdt.Rows[i][8] = (sub_counter *
int.Parse(resultdt.Rows[i][2].ToString()));
resultdt.Rows[i][9] = (AP_counter *
int.Parse(resultdt.Rows[i][2].ToString()));
resultdt.Rows[i][10] = (AT_counter *
int.Parse(resultdt.Rows[i][2].ToString()));

resultdt.Rows[i][11] = (SE_counter *
int.Parse(resultdt.Rows[i][3].ToString()));
resultdt.Rows[i][12] = (RI_counter *
int.Parse(resultdt.Rows[i][3].ToString()));
resultdt.Rows[i][13] = (A3_counter *
int.Parse(resultdt.Rows[i][3].ToString()));
resultdt.Rows[i][14] = (A5_counter *
int.Parse(resultdt.Rows[i][3].ToString()));
resultdt.Rows[i][15] = (sub_counter *
int.Parse(resultdt.Rows[i][3].ToString()));
resultdt.Rows[i][16] = (AP_counter *
int.Parse(resultdt.Rows[i][3].ToString()));
resultdt.Rows[i][17] = (AT_counter *
int.Parse(resultdt.Rows[i][3].ToString()));
}
for (int i = 0; i < resultdt.Rows.Count; i++)
{

//Treatment
SE_T_counter = (SE_T_counter +
int.Parse(resultdt.Rows[i][11].ToString()));
```

```
RI_T_counter = (RI_T_counter +
int.Parse(resultdt.Rows[i][12].ToString()));
A3_T_counter = (A3_T_counter +
int.Parse(resultdt.Rows[i][13].ToString()));
A5_T_counter = (A5_T_counter +
int.Parse(resultdt.Rows[i][14].ToString()));
sub_T_counter = (sub_T_counter +
int.Parse(resultdt.Rows[i][15].ToString()));
AP_T_counter = (AP_T_counter +
int.Parse(resultdt.Rows[i][16].ToString()));
AT_T_counter = (AT_T_counter +
int.Parse(resultdt.Rows[i][17].ToString()));

                SE_T.Text = SE_T_counter.ToString();
                RI_T.Text = RI_T_counter.ToString();
                A3_T.Text = A3_T_counter.ToString();
                A5_T.Text = A5_T_counter.ToString();
                sub_T.Text = sub_T_counter.ToString();
                AP_T.Text = AP_T_counter.ToString();
                AT_T.Text = AT_T_counter.ToString();


//control
SE_C_counter = (SE_C_counter + int.Parse(resultdt.Rows[i][4].ToString()));
RI_C_counter = (RI_C_counter + int.Parse(resultdt.Rows[i][5].ToString()));
A3_C_counter = (A3_C_counter + int.Parse(resultdt.Rows[i][6].ToString()));
A5_C_counter = (A5_C_counter + int.Parse(resultdt.Rows[i][7].ToString()));
sub_C_counter = (sub_C_counter +
int.Parse(resultdt.Rows[i][8].ToString()));
AP_C_counter = (AP_C_counter + int.Parse(resultdt.Rows[i][9].ToString()));
AT_C_counter = (AT_C_counter +
int.Parse(resultdt.Rows[i][10].ToString()));

                SE_C.Text = SE_C_counter.ToString();
                RI_C.Text = RI_C_counter.ToString();
                A3_C.Text = A3_C_counter.ToString();
                A5_C.Text = A5_C_counter.ToString();
                sub_C.Text = sub_C_counter.ToString();
                AP_C.Text = AP_C_counter.ToString();
                AT_C.Text = AT_C_counter.ToString();

            }
        }




static Int32 countChars(string STR, string Chars)
{
            var cc = STR.Replace(Chars, "");
            return (STR.Length - cc.Length) / Chars.Length;
}

private void button18_Click(object sender, EventArgs e)
```

```
{
            var chi = ChiCalculation(SE_T.Text, SE_C.Text);
            double chiPvalue_SE = Math.Round(chi.PValue, 3);
            bool Sig_SE = chi.Significant;
            SE_F.Text = chiPvalue_SE.ToString();
            SE_S.Text = TFtoSig(Sig_SE);

            chi = ChiCalculation(RI_T.Text, RI_C.Text);
            double chiPvalue_RI = Math.Round(chi.PValue, 3);
            bool Sig_RI = chi.Significant;
            RI_F.Text = chiPvalue_RI.ToString();
            RI_S.Text = TFtoSig(Sig_RI);

            chi = ChiCalculation(A3_T.Text, A3_C.Text);
            double chiPvalue_A3 = Math.Round(chi.PValue, 3);
            bool Sig_A3 = chi.Significant;
            A3_F.Text = chiPvalue_A3.ToString();
            A3_S.Text = TFtoSig(Sig_A3);

            chi = ChiCalculation(A5_T.Text, A5_C.Text);
            double chiPvalue_A5 = Math.Round(chi.PValue, 3);
            bool Sig_A5 = chi.Significant;
            A5_F.Text = chiPvalue_A5.ToString();
            A5_S.Text = TFtoSig(Sig_A5);

            chi = ChiCalculation(sub_T.Text, sub_C.Text);
            double chiPvalue_sub = Math.Round(chi.PValue, 3);
            bool Sig_sub = chi.Significant;
            sub_F.Text = chiPvalue_sub.ToString();
            sub_S.Text = TFtoSig(Sig_sub);

            chi = ChiCalculation(AP_T.Text, AP_C.Text);
            double chiPvalue_AP = Math.Round(chi.PValue, 3);
            bool Sig_AP = chi.Significant;
            AP_F.Text = chiPvalue_AP.ToString();
            AP_S.Text = TFtoSig(Sig_AP);

            chi = ChiCalculation(AT_T.Text, AT_C.Text);
            double chiPvalue_AT = Math.Round(chi.PValue, 3);
            bool Sig_AT = chi.Significant;
            AT_F.Text = chiPvalue_AT.ToString();
            AT_S.Text = TFtoSig(Sig_AT);
        }


private string TFtoSig(bool Sig)
{
            string mySig;
            if (Sig == true) mySig = "Sig."; else mySig = "Non-Sig.";
            return mySig;
}
```

```
public static ChiSquareTest ChiCalculation(string observed1, string
observed2)
{
double[] observed = { int.Parse(observed1.ToString()),
int.Parse(observed2.ToString()) };
double[] expected = { (int.Parse(observed1.ToString()) +
int.Parse(observed2.ToString())) / 2, (int.Parse(observed1.ToString()) +
int.Parse(observed2.ToString())) / 2 };
int degreesOfFreedom = 1;

var chi = new ChiSquareTest(expected, observed, degreesOfFreedom);
return chi;
}

public static ChiSquareTest ChiCalculationF(string observed1, string
observed2)
        {
double[] observed = { float.Parse(observed1.ToString()),
float.Parse(observed2.ToString()) };
double[] expected = { (float.Parse(observed1.ToString()) +
float.Parse(observed2.ToString())) / 2, (float.Parse(observed1.ToString())
+ float.Parse(observed2.ToString())) / 2 };
int degreesOfFreedom = 1;

var chi = new ChiSquareTest(expected, observed, degreesOfFreedom);
return chi;
}

private void SE_S_TextChanged(object sender, EventArgs e)
{
if (SE_S.Text == "Sig.") SE_S.ForeColor = Color.Blue; else SE_S.ForeColor
= Color.Red;
}

private void RI_S_TextChanged(object sender, EventArgs e)
{
if (RI_S.Text == "Sig.") RI_S.ForeColor = Color.Blue; else RI_S.ForeColor
= Color.Red;
}

private void A3_S_TextChanged(object sender, EventArgs e)
{
if (A3_S.Text == "Sig.") A3_S.ForeColor = Color.Blue; else A3_S.ForeColor
= Color.Red;
}


private void A5_S_TextChanged(object sender, EventArgs e)
{
if (A5_S.Text == "Sig.") A5_S.ForeColor = Color.Blue; else A5_S.ForeColor
= Color.Red;
}
private void sub_S_TextChanged(object sender, EventArgs e)
```

```
{
if (sub_S.Text == "Sig.") sub_S.ForeColor = Color.Blue; else
sub_S.ForeColor = Color.Red;
}

private void AP_S_TextChanged(object sender, EventArgs e)
{
if (AP_S.Text == "Sig.") AP_S.ForeColor = Color.Blue; else AP_S.ForeColor
= Color.Red;
}

private void AT_S_TextChanged(object sender, EventArgs e)
{
if (AT_S.Text == "Sig.") AT_S.ForeColor = Color.Blue; else AT_S.ForeColor
= Color.Red;
}

private void button22_Click(object sender, EventArgs e)
{
var chi = ChiCalculation(textBox3.Text, textBox4.Text);
double chiPvalue = Math.Round(chi.PValue, 2);
textBox5.Text = chiPvalue.ToString() + "....." + chi.Significant;
}


protected override void OnMouseWheel(MouseEventArgs e)
{
if (e.Delta > 0)
            {
                zoomInt++;
                if (zoomInt > 4)
                {
                    zoomInt = 4;
                }
                zoomPicturebox();
            }
            else if (e.Delta < 0)
            {
                zoomInt--;
                if (zoomInt < -3)
                {
                    zoomInt = -3;
                }
                zoomPicturebox();
            }
        }


private void webBrowser5_DocumentCompleted(object sender,
WebBrowserDocumentCompletedEventArgs e)
{
try
{
if (webBrowser5.Url.ToString() != "about:blank")
```

```
{
GO_WebContent = webBrowser5.Document.Body.OuterText.ToString();
                }
                string GOTerm = "Biological Process GO Terms:";
                DataTable GO_ID_dt = new DataTable();
                GO_ID_dt = ConvertToDataTable(GO_WebContent, 2);
                for (int i = 1; i < GO_ID_dt.Rows.Count; i++)
                {
GOTerm = GOTerm + "\r\n" + "- " + GO_ID_dt.Rows[i][1].ToString();}
textBox6.Text = "Gene Name:" + "\r\n" + GO_ID_dt.Rows[0][0].ToString() +
"\r\n" + GOTerm;
dataGridView2.DataSource = GO_ID_dt;

GO_ID_dt.Dispose();

}  catch   { }
}

private void dataGridView3_SelectionChanged(object sender, EventArgs e)
{
 try
{
textBox6.Text = "Please wait...";
string Draw_QueryT_ID =
dataGridView3.CurrentRow.Cells[0].Value.ToString();
string url1 = @"http://asia.ensembl.org/biomart/martservice?query=<?xml
version=" + "\"1.0\"" + " encoding=" + "\"UTF-8\"" + "?><!DOCTYPE Query>";

string url2 = @"<Query  virtualSchemaName =" + "\"default\"" + " formatter
= " + "\"TSV\"" + " header = " + "\"0\"" + " uniqueRows = " + "\"0\"" + "
count = " + "\"\"" + " datasetConfigVersion = " + "\"0.6\"" + "><Dataset
name = " + "\"" + stableID + "_gene_ensembl\"" + " interface = " +
"\"default\"" + "><Filter name = " + "\"transcript_biotype\"" + " value
=\"protein_coding\"" + "/><Filter name = " + "\"go_parent_name\"" + "
value =\"biological_process\"" + "/><Filter name = " +
"\"ensembl_transcript_id\"" + " value =\"" + Draw_QueryT_ID +
"\"/><Attribute name =" + "\"external_gene_name\"" + "/><Attribute name ="
+ "\"name_1006\"" + "/></Dataset></Query>";

string url3 = url1 + url2;
webBrowser5.Navigate(url3);
}catch { }

try
{  DrawSpliceGraph();}   catch { }  }

private void DrawSpliceGraph()
{
if (dataGridView3.RowCount < 1) return;
string Draw_QueryT_ID =
dataGridView3.CurrentRow.Cells[0].Value.ToString();

DataTable Draw_Refdt = Total_Refdt.Select("Row = '" + Draw_QueryT_ID +
"'").CopyToDataTable();
```

```
DataTable Draw_Querydt = Total_Querydt.Select("Column1 = '" +
Draw_QueryT_ID + "'").CopyToDataTable();


int Image_Len;
if (int.Parse(Draw_Refdt.Rows[Draw_Refdt.Rows.Count - 1][3].ToString()) >=
int.Parse(Draw_Querydt.Rows[Draw_Querydt.Rows.Count - 1][2].ToString()))
{
Image_Len = int.Parse(Draw_Refdt.Rows[Draw_Refdt.Rows.Count -
1][3].ToString());
}
else
{
Image_Len = int.Parse(Draw_Querydt.Rows[Draw_Querydt.Rows.Count -
1][2].ToString());            }

double Real_len = (pictureBox2.Width);

            int ExonStartPointY = 30;
            int IntronStartPointY = 35;

            int ExonHgt = 30;
            int IntronHgt = 20;
            int Startpoint = int.Parse(Draw_Refdt.Rows[0][2].ToString());

            int R_ExonStartPosition = 0;
            int R_ExonEndPosition = 0;

            int P_ExonStartPosition = 0;
            int P_ExonendPosition = 0;

            int P_Exon_Len = 0;
            int textPosition = 0;

            Bitmap bitmap = new Bitmap(1,1);
            try
            {
bitmap = new Bitmap(pictureBox2.Width, pictureBox2.Height);
            }
            catch
            {   bitmap = new Bitmap(586, 130);  }

using (Graphics myGraphic = Graphics.FromImage(bitmap))
{
myGraphic.Clear(Color.White);
int Real_SG_total_len = Image_Len -
int.Parse(Draw_Refdt.Rows[0][2].ToString());
int Pic_SG_total_len = (pictureBox2.Width - 5);
SolidBrush myBrush = new SolidBrush(Color.Blue);
string text1 = "";
for (int i = 0; i <= Draw_Refdt.Rows.Count - 1; i++)
{
```

```
R_ExonStartPosition = int.Parse(Draw_Refdt.Rows[i][2].ToString()) -
Startpoint;
R_ExonEndPosition = int.Parse(Draw_Refdt.Rows[i][3].ToString()) -
Startpoint;


P_ExonStartPosition = (R_ExonStartPosition * Pic_SG_total_len) /
Real_SG_total_len;
P_ExonendPosition = (R_ExonEndPosition * Pic_SG_total_len) /
Real_SG_total_len;
P_Exon_Len = P_ExonendPosition - P_ExonStartPosition;

Pen pen = new Pen(Color.Blue, 1);
pen.Alignment = System.Drawing.Drawing2D.PenAlignment.Inset;
myGraphic.DrawRectangle(pen, new Rectangle(P_ExonStartPosition,
ExonStartPointY, P_Exon_Len, ExonHgt));

Font font;
if (ZoomState == 2)
{
text1 = "Exon" + Draw_Refdt.Rows[i][1].ToString().Substring(4);
textPosition = P_ExonStartPosition + (P_Exon_Len / 2) - 14;

font = new Font("Arial", 7, FontStyle.Regular, GraphicsUnit.Point);
                    }
                    else
                    {
text1 = Draw_Refdt.Rows[i][1].ToString().Substring(4);
textPosition = P_ExonStartPosition + (P_Exon_Len / 2) - 5;
font = new Font("Arial", 6, FontStyle.Regular, GraphicsUnit.Point);
                    }

myGraphic.DrawString(text1, font, Brushes.Black, textPosition,
ExonStartPointY + 33);

// Transcript ID
Font font_Transcript = new Font("Arial",8, FontStyle.Regular,
GraphicsUnit.Point);
myGraphic.DrawString("Splice Graph", font_Transcript,
Brushes.LightSlateGray, 1, 0);
myGraphic.DrawString("Transcript ID: "+ Draw_Refdt.Rows[i][0].ToString(),
font_Transcript, Brushes.LightSlateGray, 1, 93);

//////SE....A5'ss...A3'ss....sub_RI

if (dataGridView3.CurrentRow.Cells[1].Value.ToString().Contains("SE(Exon"
+ Draw_Refdt.Rows[i][1].ToString().Substring(4) + ")"))
{

myGraphic.DrawString("SE(Exon" +
Draw_Refdt.Rows[i][1].ToString().Substring(4) + ")", font, Brushes.Purple,
textPosition, ExonStartPointY + 100+20);}
```

```
if
(dataGridView3.CurrentRow.Cells[1].Value.ToString().Contains("A5'SS(Exon"
+ Draw_Refdt.Rows[i][1].ToString().Substring(4) + ")"))
{
myGraphic.DrawString("A5'SS(Exon" +
Draw_Refdt.Rows[i][1].ToString().Substring(4) + ")", font, Brushes.Purple,
textPosition, ExonStartPointY + 108+20);
}

if
(dataGridView3.CurrentRow.Cells[1].Value.ToString().Contains("A3'SS(Exon"
+ Draw_Refdt.Rows[i][1].ToString().Substring(4) + ")"))
{
myGraphic.DrawString("A3'SS(Exon" +
Draw_Refdt.Rows[i][1].ToString().Substring(4) + ")", font, Brushes.Purple,
textPosition, ExonStartPointY + 118+20);

}
if
(dataGridView3.CurrentRow.Cells[1].Value.ToString().Contains("A5'&3'SS(Exo
n" + Draw_Refdt.Rows[i][1].ToString().Substring(4) + ")"))
{
myGraphic.DrawString("A5'&3'SS(Exon" +
Draw_Refdt.Rows[i][1].ToString().Substring(4) + ")", font, Brushes.Purple,
textPosition, ExonStartPointY + 100+20);

}

if (dataGridView3.CurrentRow.Cells[1].Value.ToString().Contains("RI(Exon"
+ Draw_Refdt.Rows[i][1].ToString().Substring(4) + ")"))
{

if
(dataGridView3.CurrentRow.Cells[1].Value.ToString().Contains("sub_RI(Exon"
+ Draw_Refdt.Rows[i][1].ToString().Substring(4) + ")"))
{

if
(dataGridView3.CurrentRow.Cells[1].Value.ToString().Contains("SE/sub_RI(Ex
on" + Draw_Refdt.Rows[i][1].ToString().Substring(4) + ")"))
{
myGraphic.DrawString("SE/sub_RI(Exon" +
Draw_Refdt.Rows[i][1].ToString().Substring(4) + ")", font, Brushes.Purple,
textPosition + 20, ExonStartPointY + 102+20);

}
else myGraphic.DrawString("sub_RI(Exon" +
Draw_Refdt.Rows[i][1].ToString().Substring(4) + ")", font, Brushes.Purple,
textPosition + 20, ExonStartPointY + 102+20);

}
else myGraphic.DrawString("RI(Exon" +
Draw_Refdt.Rows[i][1].ToString().Substring(4) + ")", font, Brushes.Purple,
textPosition, ExonStartPointY + 102+20);}}
```

```
for (int j = 0; j <= Draw_Refdt.Rows.Count - 2; j++)
{
R_ExonStartPosition = int.Parse(Draw_Refdt.Rows[j][3].ToString()) -
Startpoint;
R_ExonEndPosition = int.Parse(Draw_Refdt.Rows[j + 1][2].ToString()) -
Startpoint;

P_ExonStartPosition = (R_ExonStartPosition * Pic_SG_total_len) /
Real_SG_total_len;

P_ExonendPosition = (R_ExonEndPosition * Pic_SG_total_len) /
Real_SG_total_len;
P_Exon_Len = P_ExonendPosition - P_ExonStartPosition;

myBrush = new SolidBrush(Color.Gray);
myGraphic.FillRectangle(myBrush, new Rectangle(P_ExonStartPosition,
IntronStartPointY, P_Exon_Len, IntronHgt));
}


for (int z = 0; z <= Draw_Querydt.Rows.Count - 2; z++)
{
if (int.Parse(Draw_Querydt.Rows[z + 1][1].ToString()) <=
int.Parse(Draw_Refdt.Rows[Draw_Refdt.Rows.Count - 1][3].ToString()))

{
R_ExonStartPosition = int.Parse(Draw_Querydt.Rows[z][2].ToString()) -
Startpoint;
R_ExonEndPosition = int.Parse(Draw_Querydt.Rows[z + 1][1].ToString()) -
Startpoint;

P_ExonStartPosition = (R_ExonStartPosition * Pic_SG_total_len) /
Real_SG_total_len;
P_ExonendPosition = (R_ExonEndPosition * Pic_SG_total_len) /
Real_SG_total_len;
P_Exon_Len = P_ExonendPosition - P_ExonStartPosition;

//Create points that define line.
Point point1 = new Point(P_ExonStartPosition, ExonStartPointY + 4);
Point point3 = new Point(P_ExonStartPosition + (P_Exon_Len / 2),
ExonStartPointY - 13);
Point point4 = new Point(P_ExonendPosition, ExonStartPointY + 4);


float[] dashValues = { 4, 2 };
Pen dashPen = new Pen(Color.Gray, 1);
dashPen.DashPattern = dashValues;

myGraphic.DrawLines(dashPen, new Point[] { point1, point3, point4 });
}
}

for (int q = 0; q <= Draw_Querydt.Rows.Count - 1; q++)
{
```

```
R_ExonStartPosition = int.Parse(Draw_Querydt.Rows[q][1].ToString()) -
Startpoint;
R_ExonEndPosition = int.Parse(Draw_Querydt.Rows[q][2].ToString()) -
Startpoint;


P_ExonStartPosition = (R_ExonStartPosition * Pic_SG_total_len) /
Real_SG_total_len;
P_ExonendPosition = (R_ExonEndPosition * Pic_SG_total_len) /
Real_SG_total_len;

P_Exon_Len = P_ExonendPosition - P_ExonStartPosition;

myBrush = new SolidBrush(Color.Blue);
myGraphic.FillRectangle(myBrush, new Rectangle(P_ExonStartPosition,
ExonStartPointY + 70+20, P_Exon_Len, ExonHgt));


/// AP...AT
Font font = new Font("Arial", 7, FontStyle.Regular, GraphicsUnit.Point);
if ((dataGridView3.CurrentRow.Cells[1].Value.ToString().Contains("AP")) &
q == 0)
{
myGraphic.DrawString("AP", font, Brushes.Purple, P_ExonStartPosition,
ExonStartPointY + 57+20);}

if ((dataGridView3.CurrentRow.Cells[1].Value.ToString().Contains("AT")) &
q == Draw_Querydt.Rows.Count - 1)
{
myGraphic.DrawString("AT", font, Brushes.Purple, P_ExonendPosition - 15,
ExonStartPointY + 57+20);
}}

for (int p = 0; p <= Draw_Querydt.Rows.Count - 2; p++)
{
R_ExonStartPosition = int.Parse(Draw_Querydt.Rows[p][2].ToString()) -
Startpoint;
R_ExonEndPosition = int.Parse(Draw_Querydt.Rows[p + 1][1].ToString()) -
Startpoint;

P_ExonStartPosition = (R_ExonStartPosition * Pic_SG_total_len) /
Real_SG_total_len;

P_ExonendPosition = (R_ExonEndPosition * Pic_SG_total_len) /
Real_SG_total_len;
P_Exon_Len = P_ExonendPosition - P_ExonStartPosition;

myBrush = new SolidBrush(Color.LightGray);
myGraphic.FillRectangle(myBrush, new Rectangle(P_ExonStartPosition,
IntronStartPointY + 70+20, P_Exon_Len, IntronHgt));                   }
}
this.pictureBox2.Image = bitmap;
}
```

```
int zoomInt = 0;
private void Open_btn_Click(object sender, EventArgs e)
{
Image image;

// open file dialog
OpenFileDialog open = new OpenFileDialog();
// image filters
open.Filter = "Image Files(*.jpg; *.jpeg; *.gif; *.bmp)|*.jpg; *.jpeg;
*.gif; *.bmp";
if (open.ShowDialog() == DialogResult.OK)
            {
                image = Image.FromFile(open.FileName);
                pictureBox2.Image = image;
            }
}

void pictureBox2_MouseWheel(object sender, MouseEventArgs e)
{
label1.Text = "64654564646";
            if (e.Delta > 0)
            {
                zoomInt++;
                if (zoomInt > 4)
                {
                    zoomInt = 4;
                }
                zoomPicturebox();
            }
            else if (e.Delta < 0)
            {
                zoomInt--;
                if (zoomInt < -3)
                {
                    zoomInt = -3;
                }
                zoomPicturebox();
            }
}

public void zoomPicturebox()
{
switch (zoomInt)
{
                case -3:
                    this.pictureBox2.Width -= 210;
                    break;
                case -2:
                    this.pictureBox2.Width -= 155;
                    break;
                case -1:
                    this.pictureBox2.Width -= 65;
                    break;
                case 0:
```

```
                    pictureBox2.Size = new Size(850, 1100);
                    break;
                case 1:
                    this.pictureBox2.Width += 75;
                    break;
                case 2:
                    this.pictureBox2.Width += 150;
                    break;
                case 3:
                    this.pictureBox2.Width += 175;
                    break;
                case 4:
                    this.pictureBox2.Width += 200;
                    break;
}

pictureBox2.Refresh(); //Helps causing pictures from getting pixialated:
Forces the control to invalidate its client area and immediately redraw
itself and any child controls
DrawSpliceGraph();
}

private void button23_Click(object sender, EventArgs e)
{
            pictureBox2.Height = pictureBox2.Height + 100;
            pictureBox2.Width = pictureBox2.Width + 100;
            DrawSpliceGraph();
}

private void panel8_SizeChanged(object sender, EventArgs e)
{
DrawSpliceGraph();
}

private void pictureBox2_MouseEnter(object sender, EventArgs e)
{
pictureBox2.Focus();
}

int ZoomState = 1;
private void pictureBox2_Click(object sender, EventArgs e)
{
if (this.pictureBox2.Width == 10000)
            {
                ZoomState = 1;
                pictureBox2.Width = panel8.Width - 10;
                pictureBox2.Height = 160;
            }
else if (this.pictureBox2.Width == panel8.Width-10)
            {
                ZoomState = 2;
                pictureBox2.Width = 10000;
                pictureBox2.Height = 160;
            }
```

```
}

private void dataGridView3_CellDoubleClick(object sender,
DataGridViewCellEventArgs e)
{
            rB1.Checked = true;
            button4_Click_1(null, null);
            dataGridView3_CellDoubleClick(null, null);
}

private void button24_Click(object sender, EventArgs e)
{
string Draw_QueryT_ID =
dataGridView3.CurrentRow.Cells[0].Value.ToString();
DataTable Draw_Refdt = Total_Refdt.Select("Row = '" + Draw_QueryT_ID +
"'").CopyToDataTable();
DataTable Draw_Querydt = Total_Querydt.Select("Column1 = '" +
Draw_QueryT_ID + "'").CopyToDataTable();
}

private void dataGridView3_RowsAdded(object sender,
DataGridViewRowsAddedEventArgs e)
{
try
{DrawSpliceGraph();   }
catch { }
}

public static string Ereader(string path)
{
            string text = System.IO.File.ReadAllText(path);
            return text;
}

public DataTable ConvertToDataTable_text(string filePath, int
numberOfColumns)
{
DataTable tbl = new DataTable();
for (int col = 0; col < numberOfColumns; col++)
tbl.Columns.Add(new DataColumn("Column" + (col + 1).ToString()));
string[] lines = System.IO.File.ReadAllLines(filePath);
foreach (string line in lines)
{
var cols = line.Split('\t');
DataRow dr = tbl.NewRow();
for (int cIndex = 0; cIndex < numberOfColumns; cIndex++)
{
try
{  dr[cIndex] = cols[cIndex]; }
catch { }
}
tbl.Rows.Add(dr); }
return tbl;
}
```

```
private void button13_Click_1(object sender, EventArgs e)
{
            PathText = openFile();
            DataTable Pre_GXFdt = ConvertToDataTable_text(PathText, 10);

            DataTable GXFdt =makeDataTable(1) ;
            if (radioButton1.Checked == true)
            {
                for (int x = 0; x < Pre_GXFdt.Rows.Count; x++)
                    if (Pre_GXFdt.Rows[x][2].ToString() == "transcript")
                    {
                        DataRow dr = GXFdt.NewRow();
                        string s = Pre_GXFdt.Rows[x][9].ToString();
                        int index = s.IndexOf("ENST");
                        if (index != -1)
                        {
                            dr[0] = s.Substring(index , 15);
                        }
                        GXFdt.Rows.Add(dr);
                    }
            }

if (radioButton2.Checked == true)
{
for (int x = 0; x < Pre_GXFdt.Rows.Count; x++)
if (Pre_GXFdt.Rows[x][2].ToString() == "transcript")
{
                        DataRow dr = GXFdt.NewRow();
                        string s = Pre_GXFdt.Rows[x][8].ToString();
                        int index = s.IndexOf("ENST");
if (index != -1)
{
dr[0] = s.Substring(index ,  15);
}
GXFdt.Rows.Add(dr);
}
}

dataGridView1.DataSource = GXFdt;
Inputdt = GXFdt;

}


private void StatBox_CheckedChanged(object sender, EventArgs e)
{
if (StatBox.Checked == true)
            {
                groupBox9.Visible = true;
                try
                {
                    if (Activity == "stat")
                    {
```

```
                        groupBox9.Visible = true;
                        groupBox13.Visible = true;
                        button14_Click(null, null);
                        button17_Click(null, null);
                        button18_Click(null, null);
                    }
                    else
                    {
                        groupBox9.Visible = false;
                        groupBox13.Visible = false;
                    }
                }
                catch {  return;   }
            }
            else
                if (StatBox.Checked == false)
            {
                groupBox9.Visible = false;
                groupBox13.Visible = false;
            }

        }

private void dataGridView1_SelectionChanged(object sender, EventArgs e)
{
if (dataGridView1.RowCount > 1)
{rB2.Checked = true;}

}

private void pictureBox2_DoubleClick(object sender, EventArgs e)
{
ZoomState = 1;
pictureBox2.Width = 586;
pictureBox2.Height = 150;
}

private void Form1_FormClosing(object sender, FormClosingEventArgs e)
{
Environment.Exit(Environment.ExitCode);
}


private void button17_Click_1(object sender, EventArgs e)
{
SaveFileDialog dialog = new SaveFileDialog();
dialog.DefaultExt = "jpg";
dialog.Filter = "jpg files (*.jpg)|*.jpg|All files (*.*)|*.*";
if (dialog.ShowDialog() == DialogResult.OK)
{
int width = Convert.ToInt32(pictureBox2.Width);
int height = Convert.ToInt32(pictureBox2.Height);
Bitmap bmp = new Bitmap(width, height);
pictureBox2.DrawToBitmap(bmp, new Rectangle(0, 0, width, height));
```

```
bmp.Save(dialog.FileName, ImageFormat.Jpeg);
}
}

private void button14_Click_1(object sender, EventArgs e)
{
ChiCalculation("45", "2");
}

private void chiSquerToolStripMenuItem_Click(object sender, EventArgs e)
{           Activity = "stat";
            button1_Click(null, null);
            i = 0;
            dataGridView3.DataSource = null;
            try
            {
                Total_Refdt.Dispose();
                Total_Querydt.Dispose();
            }   catch { }
        }
private void excelToolStripMenuItem_Click(object sender, EventArgs e)
{           Activity = "simple";
            button1_Click(null, null);
            i = 0;
            dataGridView3.DataSource = null;
try
{         resultdt.Clear();
           resultdt.Dispose();
           dataGridView3.DataSource = null;
           Total_Refdt.Dispose();
           Total_Querydt.Dispose();
} catch { }
}

private void gTFToolStripMenuItem_Click(object sender, EventArgs e)
        {
            Activity = "GTF";
            radioButton1.Checked = true;
            button13_Click_1(null, null);
        }

private void gFF3ToolStripMenuItem_Click(object sender, EventArgs e)
        {
            Activity = "GFF3";
            radioButton2.Checked = true;
            button13_Click_1(null, null);
        }

private void Form1_ResizeEnd(object sender, EventArgs e)
        {
            pictureBox2.Height = panel8.Height-10;
            pictureBox2.Width = panel8.Width-10;
        }
private void Form1_Resize(object sender, EventArgs e)
```

```
        {
            pictureBox2.Height = panel8.Height-10;
            pictureBox2.Width = panel8.Width-10;
            DrawSpliceGraph();

            groupBox9.Height = groupBox12.Height;
            groupBox9.Width = groupBox12.Width;
            groupBox9.Left = groupBox12.Left;
            groupBox9.Top = groupBox12.Top;
        }

private void downloadManualToolStripMenuItem_Click(object sender,
EventArgs e)
        { Process.Start(@"Practical_guide.pdf");        }

private void aboutToolStripMenuItem_Click(object sender, EventArgs e)
        {
            AboutBox1 ab = new AboutBox1();
            ab.Show();
        }
}}
```

Supplementary Note 3:
Sample XML file content to retrieve genomic coordinates of query transcript exons

<?xml version="1.0" encoding="UTF-8"?>

<!DOCTYPE Query>

<Query datasetConfigVersion="0.6" count="" uniqueRows="0" header="0" formatter="TSV" virtualSchemaName="default"><Dataset interface="default" name="hsapiens_gene_ensembl"><Filter name="ensembl_transcript_id" value="ENST00000565123"/><Attribute name="ensembl_gene_id"/><Attribute name="ensembl_transcript_id"/><Attribute name="exon_chrom_start"/><Attribute name="exon_chrom_end"/><Attribute name="strand"/></Dataset></Query>

Supplementary Note 4:
Practical guide

SpliceDetector: a software for detection of alternative splicing events in human and model organisms directly from transcript IDs

Practical guide

Background

SplicDetector software is a Windows application that identifies the Alternative Splice events through Splice Graph construction for every transcript of interest, using known transcripts annotation in Ensembl database. This tool takes the Ensembl transcript IDs as input data and performs the following analysis:

1. Identifying Alternative Splicing events in transcripts in Single/Multiple form
2. Presenting gene information and a graph view of transcript splicing in comparison with the constructed splice graph
3. Statistical analysis of differential splicing alteration in experimental transcripts

Platform(s) and requirement

The SpliceDetector software is compatible with Windows 7, 8, 8.1, and 10 and requires .NET Framework 4.5 component on the client computer.

Example datasets

Data for testing can be found here and also is supplied as following supplementary data files:

1. file S6 for input data in multiple form searching
2. file S7 for GTF input data format
3. file S8for GFF3 input data format
4. file S9 for statistical analysis

Figure 1 shows an overall view of the application. The left side is for input data entry and setting of analysis parameters. SplicDetector accepts transcript IDs individually for single searching in a textbox in the top left corner as well as a set of transcripts for multiple searching in the provided grid.

The middle section shows analysis results including transcripts AS events as a table and a graph view of the current transcript (selected transcript in the table) in the bottom.

On the right side of software window, Event Log section provides some information about analysis performance. Gene Information section at bottom right corner gives some knowledge about genes associated to transcripts and relevant gene ontology terms.

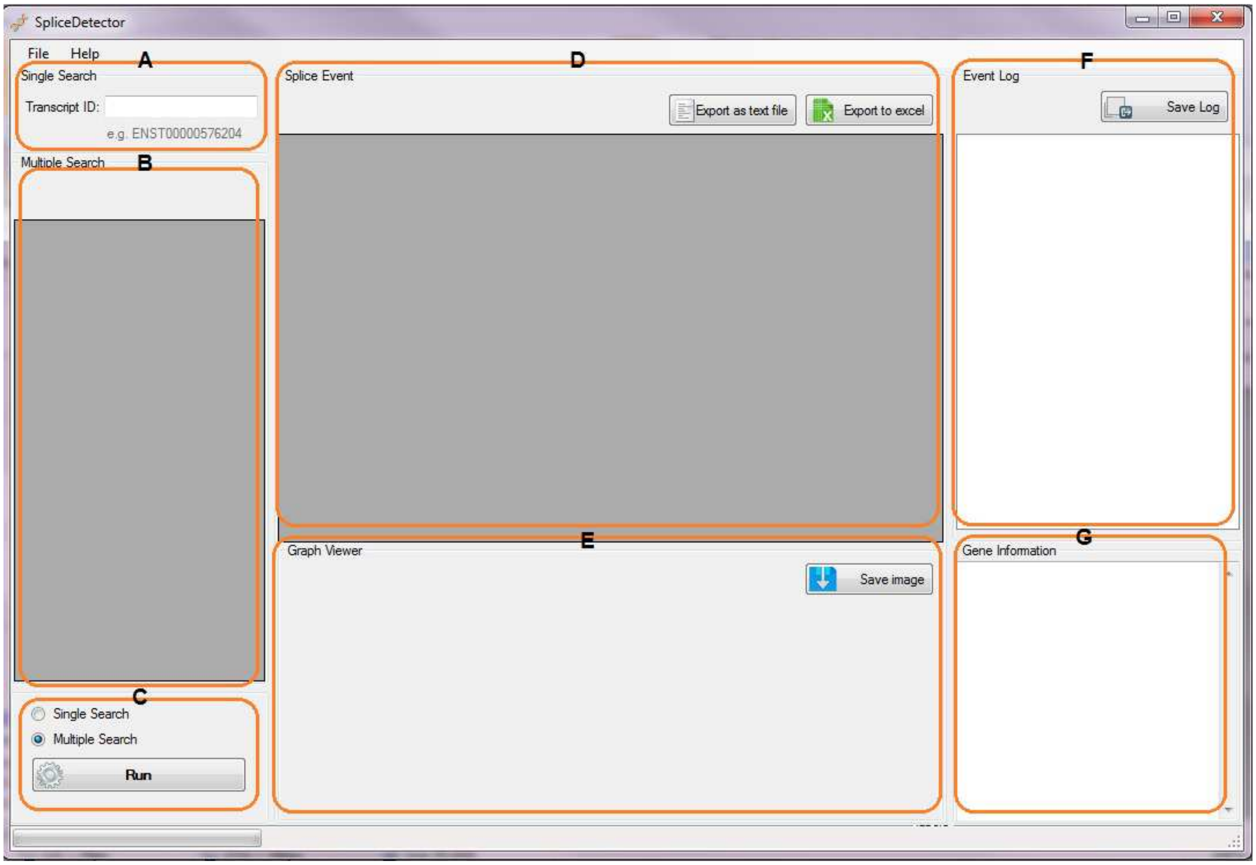

Figure 1: **An overview of SpliceDetector tool . A)**a textbox to import data for single searching **B)** a grid to import a set of transcripts for multiple searching **C)** options for setting parameters and running the analysis **D)** a grid for showing analysis results including transcripts IDs and extracted AS events as a table **E)** Graph viewer for presenting a view of current transcript exons in comparison with corresponded splice graph **F)** Event Log section for providing information about analysis performance during the application working **G)** a section for providing some information about corresponding genes to transcripts and related gene ontology terms.

Data entry and performing analysis

There are two methods for entering transcript IDs as input. The first one is manually entering which can be done by 'Single Search' type and the second one is entering transcript IDs as a transcript set which has been provided as 'Multiple Search' type.

I. Single search:
There is a specified textbox for entering single Ensembl transcript ID. In this case, the transcript ID is entered manually in a textbox on the left top of the application window. Then, the 'Single Search' method is selected and running starts by clicking on 'Run' button (Fig.2).

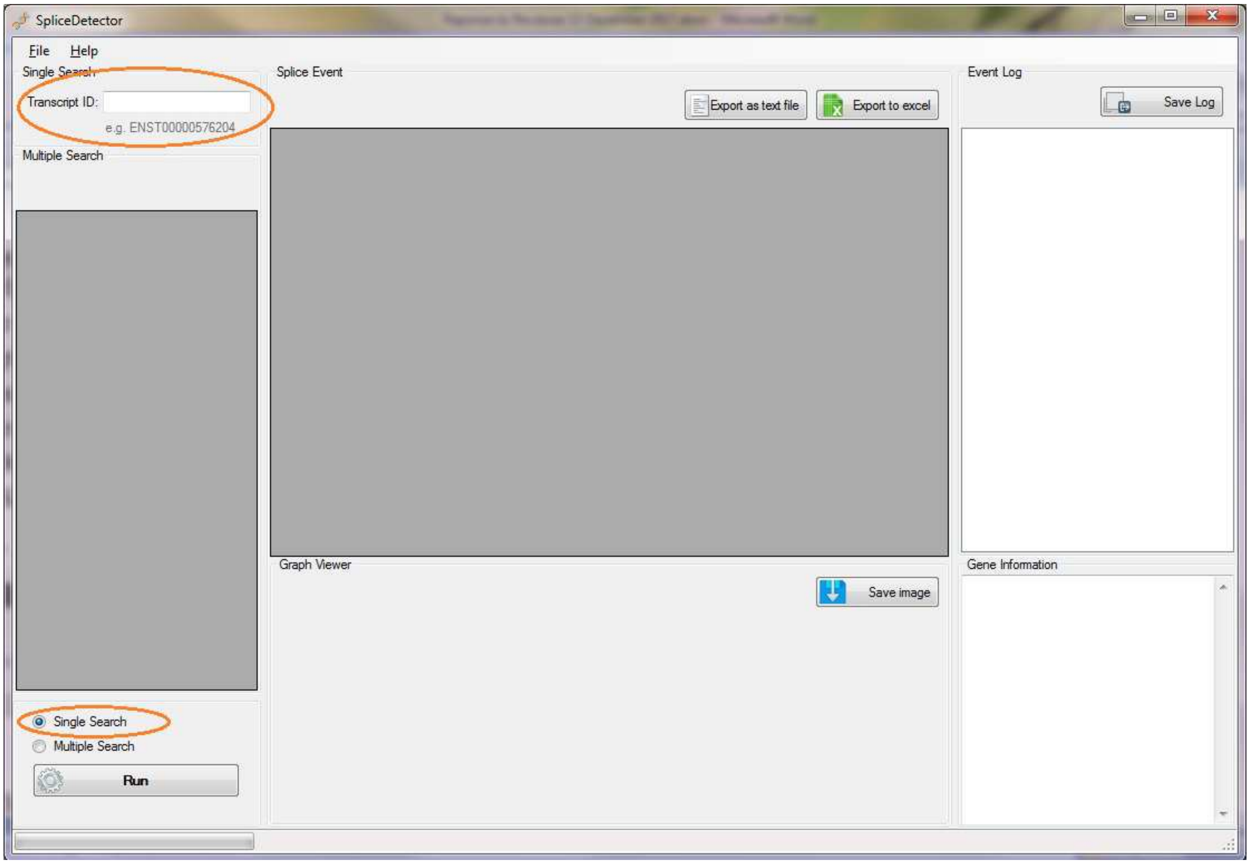

Figure 2:**Single search application of SpliceDetector.** A transcript Ensembl ID is entered in a textbox manually and the option of 'Single Search' is selected. Then 'Run' button can be clicked.

II. Multiple search:
For multiple searching, a set of transcript IDs is entered in splicing identification process (Fig.3).The provided grid, on the left side of application window, shows the list of transcript IDs after entering. Users can import data for Multiple search, by selecting **Import data** option from **File** menu(Fig.4.a). Then, a new explorer window (Fig.4.b) is opened to browse the accessing path of the input data.

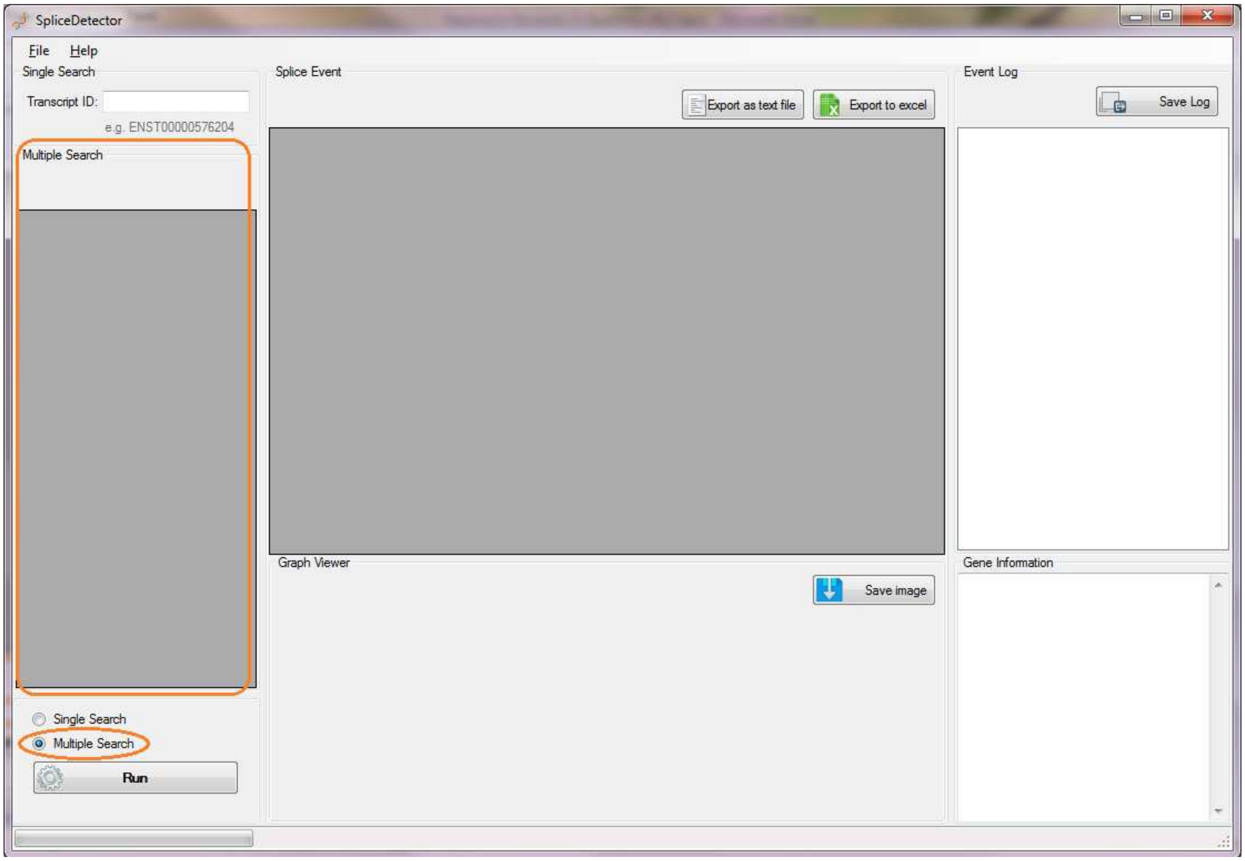

Figure 3:**Multiple search application of SpliceDetector.** An excel file including Ensembl transcript IDs is entered using dialog form and then the option of 'Multiple Search' is selected.

The application accepts data in Microsoft excel, GTF, and GFF3 formats.

a) 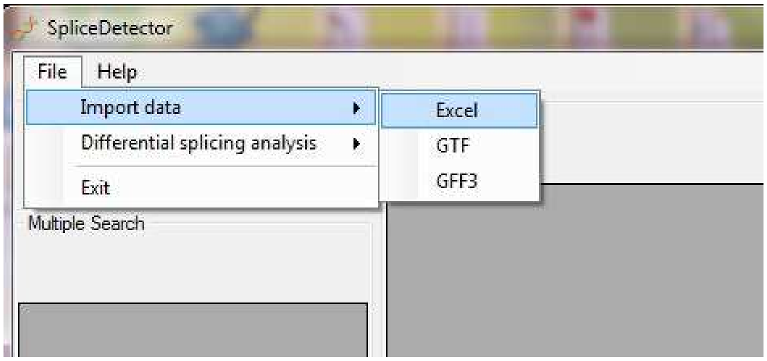

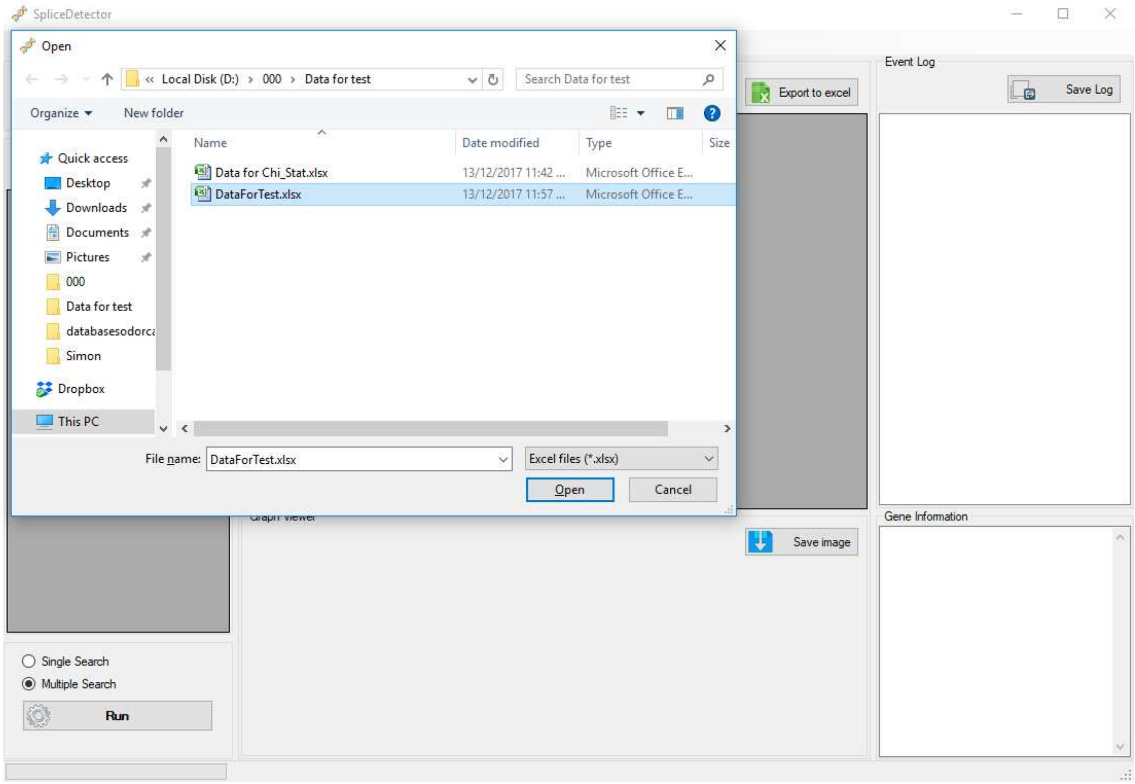

b)

Figure 4: **Data Import for Multiple Search.** a) For multiple search, the input data is imported using 'File' menu. b) The input data including Ensembl transcripts IDs in Excel, GTF, and GFF3 formats is entered to application using 'Open dialog box'.

For importing transcripts set in Excel format as input data ,it is necessary that the sheet of excel file containing transcript IDs to be named as 'Sheet1' and the data column should contain an arbitrary column header (Fig.5). There is no extra setting for importing data in GTF ad GFF3 formats. In the next step, the 'Multiple Search' method is selected and running is started by clicking on 'Run' button. The analysis results are presented in a table including transcript IDs and extracted alternative splicing events. A report of analysis steps performance is accessible in Event Log section.

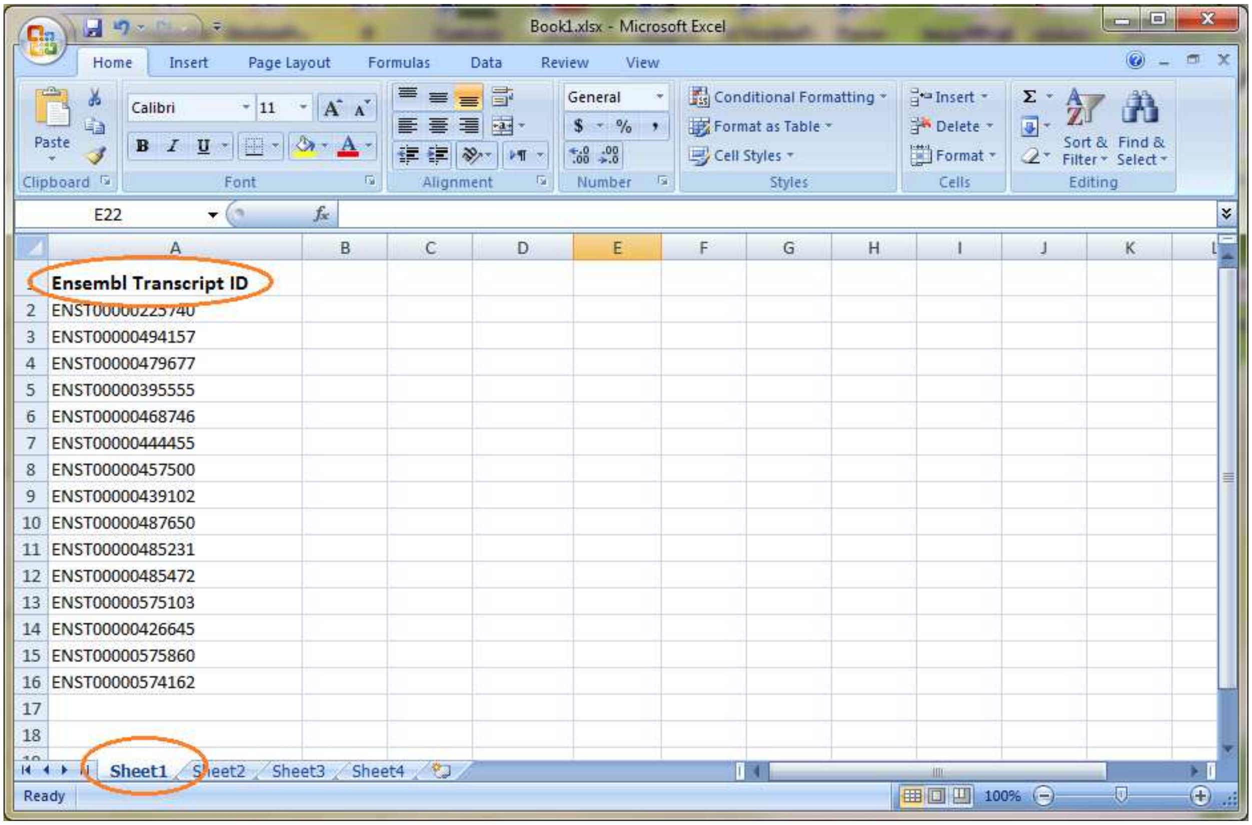

Figure 5.**Example of importing data in Excel file for Multiple Search**

Graph viewer at the bottom of the application window shows the query transcript exons as well as the corresponded Splice Graph that provides an understanding of splice sites and alternative splicing events. Some information about the transcript, including the associated gene name and gene ontology (GO) are provided at Gene Information section as well. By selecting the transcript ID of interest in the table (mouse clicking), the graph view and gene information appear in the provided sections (Fig.6).

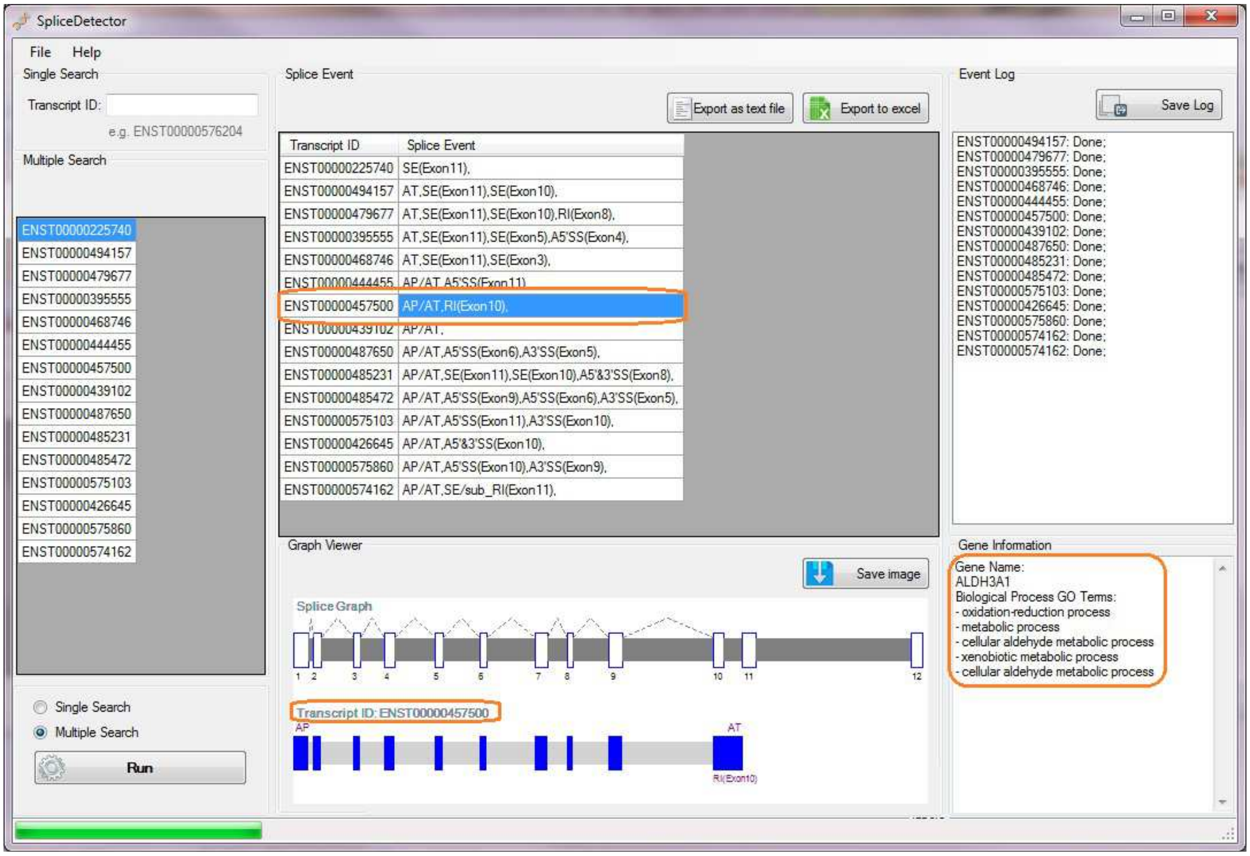

Figure 6. **Graph Viewer and Gene Information section.** The presented information and graphs are related to the selected transcript in the splice event table.

Differential splicing analysis

Alternative splicing plays a major role in gene expression regulation due to its effect on expanding proteome diversity[1]. Therefore, to discover the treatment effect on cell activities through the alternative splicing, a statistical analysis of AS events of transcripts can be performed to evaluate the effect of treatment on AS events alteration before and after the treatment. In a comparison between an experimental group and a control group, the number of AS events of each transcript before and after treatment can be calculated by AS events number of that transcript multiply by its unique mapped reads for both control and treated samples. The count of unique mapped reads to transcript is considered as an effective read count for AS events to avoid read mapping errors and prevent false positive outcomes[2].

To determine the significance of alternative splicing alteration (comparison of control group to experimental group), the unique read counts of transcripts before (control sample) and after (treated sample) the treatment need to be imported along with transcript IDs in an excel format with three columns (Fig.7). The application performs a Chi-square Goodness of Fit statistical test[3] to calculate significance of alteration rates between the Experimental Group and Control Group using the estimated number of alternative splicing events.

The provided data for test (supplementary file S9) is a set of differentially expressed transcripts in response to Genistein treatment (the soy isoflavone metabolite). This differentially expressed transcripts list was generated from MCF-7 breast cancer cell line RNA-Seq data (FASTQ files) downloaded from GEO database under accession number GSE56066[4].

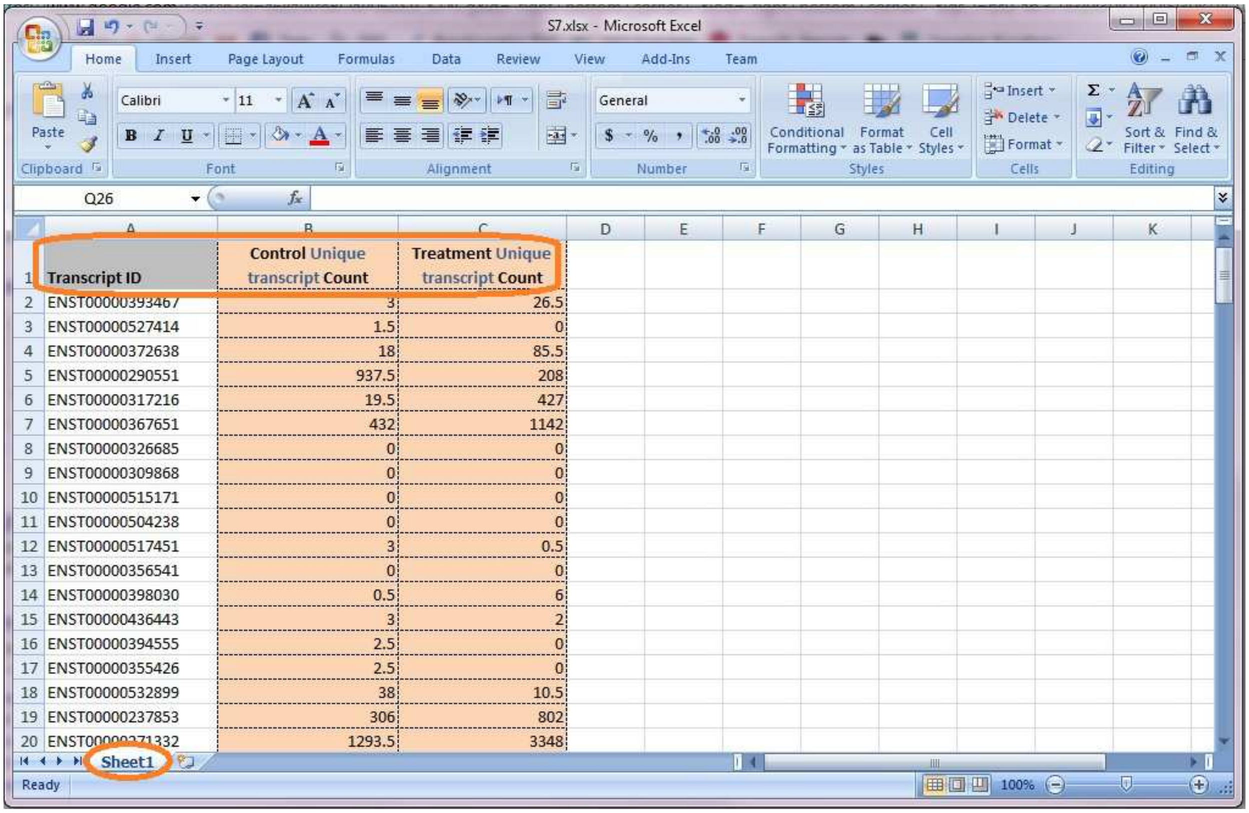

Figure 7.**Importing data for differential splicing analysis**. Input data for differential splicing analysis needs to be outcome of differential expression experiments. The input data in excel format includes three columns: transcripts IDs, unique read count of transcripts in control sample (before the treatment), and unique read count of transcripts in treated sample (after the treatment). The sheet of excel file including transcript IDs needs to be named as 'Sheet1'.

Steps to perform differential splicing analysis

1. From the File menu(Fig.8.a), select:
   File → Differential splicing analysis →Chi-square Goodness of Fit test
2. Browse the path for the excel file of input data including unique read count values of transcripts before and after the treatment
3. Run the alternative splicing analysis completely by clicking on 'Run' button
4. Check the 'Stat' check box(Fig.8.b)

a)

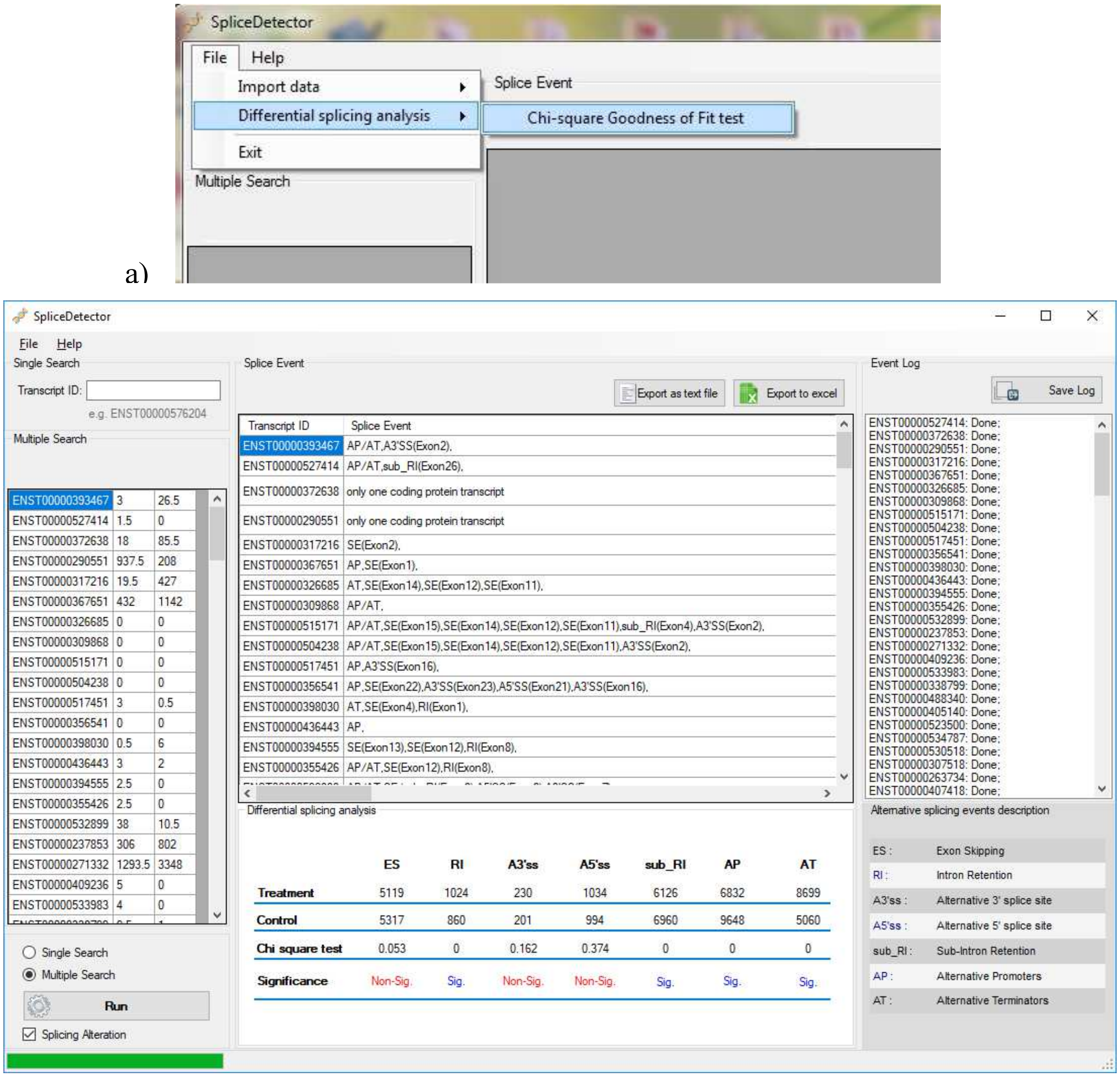

b)

Figure 8:**Statistical analysis of splicing events alteration in splicing variants before and after the treatment**

Exporting the results

The application gives the user the options of exporting results in both of text and Microsoft excel format(Fig.9).

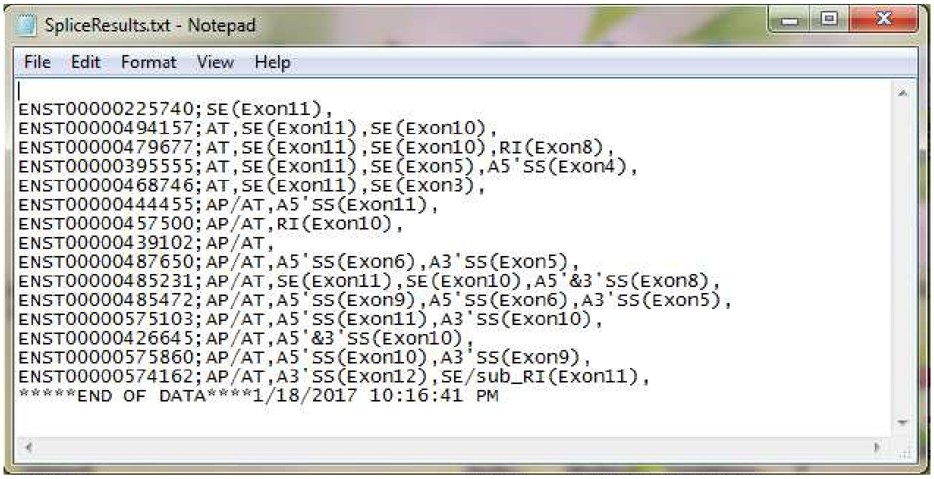

a)

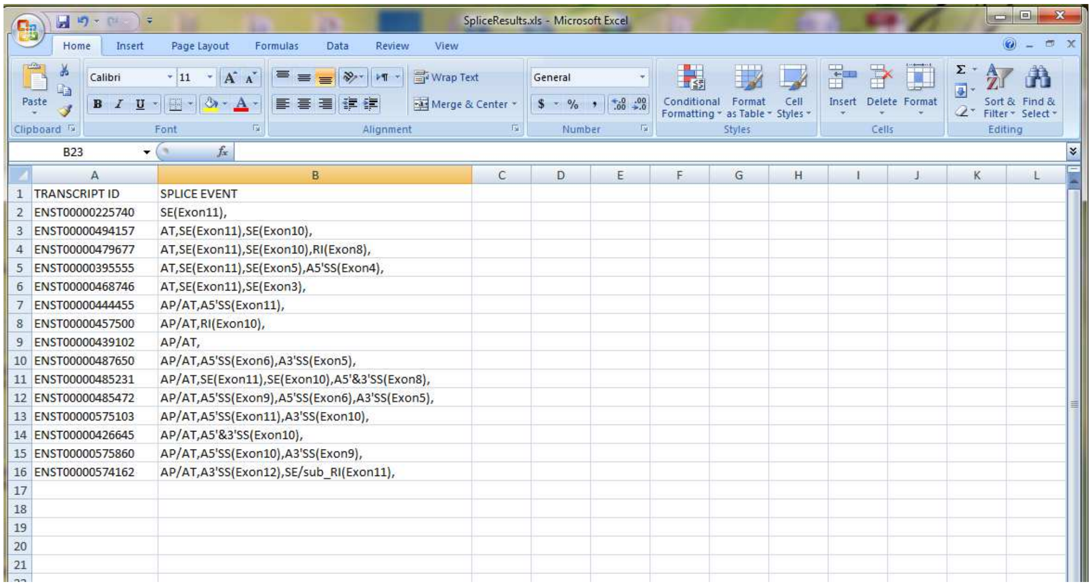

b)

Figure 9:**Exporting the results of SpliceDetector.** a) text format, b) excel format.

In addition, the graph view of transcripts and their corresponded splice graphs can be exported using 'Save Image' button(Fig.10).

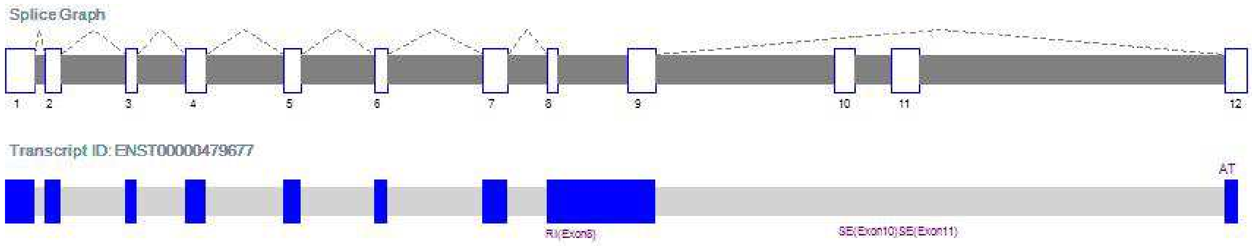

Figure 10.**An example image of a transcript with alternative splicing in comparison with corresponded splice graph.**

References:

1 Magen, A. & Ast, G. The importance of being divisible by three in alternative splicing. *Nucleic acids research* **33**, 5574-5582, doi:10.1093/nar/gki858 (2005).

2 Pyrkosz, A. B., Cheng, H. H. & Brown, C. T. (arxiv.org. arXiv:1303.2411v1, 2013).

3 McDonald, J. H. *Handbook of Biological Statistics*. (Sparky House Publishing, 2014).

4 Gong, P. *et al.* Transcriptomic analysis identifies gene networks regulated by estrogen receptor alpha (ERalpha) and ERbeta that control distinct effects of different botanical estrogens. *Nuclear receptor signaling* **12**, e001, doi:10.1621/nrs.12001 (2014).

Supplementary Note 5:
Application executive and essential files download link

Platform(s) and requirement
The SpliceDetector software is compatible with Windows 7, 8, 8.1, and 10 and requires .NET Framework 4.5 component on the client computer.

Google Drive link:
https://drive.google.com/open?id=1dlXKzbvxOH3A85_DVR__V2eI5s16-llv

Dropbox link:
https://www.dropbox.com/s/j5o0og159ig6tej/SpliceDetector%20Executable%20File.rar?dl=0

Supplemental Tables:

Start Position	End Position	Strand direction
83953239	83953370	+1
83957640	83957738	+1
83959260	83959396	+1
83960569	83960760	+1
83960981	83961253	+1

Supplementary Table ST1: Genomic coordinates of Query Transcript ID

Exon_ID	Start Position	End Position
ENSE00002594865	83948282	83948332
ENSE00003558096	83948814	83948901
ENSE00003577626	83949037	83949107
ENSE00003559276	83949403	83949506
ENSE00002587588	83953262	83953370
ENSE00002596654	83957640	83957650
ENSE00003514176	83949067	83949506
ENSE00003637052	83957640	83957738
ENSE00001013991	83959260	83959396
ENSE00000945798	83960569	83960760
ENSE00000945799	83960981	83961072
ENSE00002581631	83965062	83966331
ENSE00003660841	83949067	83949506
ENSE00001334593	83951128	83951287
ENSE00003686092	83957640	83957738
ENSE00001013991	83959260	83959396
ENSE00000945798	83960569	83960760
ENSE00000945799	83960981	83961072
ENSE00001391041	83965062	83966332
ENSE00001514774	83953222	83953370
ENSE00003637052	83957640	83957738
ENSE00001013991	83959260	83959396
ENSE00000945798	83960569	83960760
ENSE00000945799	83960981	83961072
ENSE00001391041	83965062	83966332
ENSE00002606461	83953239	83953370
ENSE00003637052	83957640	83957738
ENSE00001013991	83959260	83959396
ENSE00000945798	83960569	83960760
ENSE00002602349	83960981	83961253
ENSE00002627817	83956988	83957121
ENSE00003637052	83957640	83957738
ENSE00001013991	83959260	83959396
ENSE00002621390	83960569	83960732

Supplementary Table ST2: Genomic coordinates of all known exons related to the detected Gene ID

Position	Start/End	Exon_ID	frequency
83948282	Start	ENSE00002594865	1
83948332	End	ENSE00002594865	1
83948814	Start	ENSE00003558096	1
83948901	End	ENSE00003558096	1
83949037	Start	ENSE00003577626	1
83949067	Start	ENSE00003514176	2
83949107	End	ENSE00003577626	1
83949403	Start	ENSE00003559276	1
83949506	End	ENSE00003559276	3
83951128	Start	ENSE00001334593	1
83951287	End	ENSE00001334593	1
83953222	Start	ENSE00001514774	1
83953239	Start	ENSE00002606461	1
83953262	Start	ENSE00002587588	1
83953370	End	ENSE00002587588	3
83956988	Start	ENSE00002627817	1
83957121	End	ENSE00002627817	1
83957640	Start	ENSE00002596654	6
83957650	End	ENSE00002596654	1
83957738	End	ENSE00003637052	5
83959260	Start	ENSE00001013991	5
83959396	End	ENSE00001013991	5
83960569	Start	ENSE00000945798	5
83960732	End	ENSE00002621390	1
83960760	End	ENSE00000945798	4
83960981	Start	ENSE00000945799	4
83961072	End	ENSE00000945799	3
83961253	End	ENSE00002602349	1
83965062	Start	ENSE00002581631	3
83966331	End	ENSE00002581631	1
83966332	End	ENSE00001391041	2

Supplementary Table ST3. Algorithm implementation: All start and end points of all exons were collected in a pool; Then the collected start and end points of mentioned exons were sorted and their frequencies were measured.

Position	Start/End	Exon_ID	frequency	SpliceGraph Including	Algorithm implementation
83948282	Start	ENSE00002594865	1	-	
83948332	End	ENSE00002594865	1	-	
83948814	Start	ENSE00003558096	1	-	
83948901	End	ENSE00003558096	1	-	
83949037	Start	ENSE00003577626	1	-	
83949067	Start	ENSE00003514176	2	✓	*
83949107	End	ENSE00003577626	1	✓	
83949403	Start	ENSE00003559276	1	✓	
83949506	End	ENSE00003559276	3	✓	
83951128	Start	ENSE00001334593	1	-	
83951287	End	ENSE00001334593	1	-	
83953222	Start	ENSE00001514774	1	✓	**
83953239	Start	ENSE00002606461	1	-	
83953262	Start	ENSE00002587588	1	-	
83953370	End	ENSE00002587588	3	✓	
83956988	Start	ENSE00002627817	1	-	
83957121	End	ENSE00002627817	1	-	
83957640	Start	ENSE00002596654	6	✓	***
83957650	End	ENSE00002596654	1	-	
83957738	End	ENSE00003637052	5	✓	
83959260	Start	ENSE00001013991	5	✓	***
83959396	End	ENSE00001013991	5	✓	
83960569	Start	ENSE00000945798	5	✓	***
83960732	End	ENSE00002621390	1	-	
83960760	End	ENSE00000945798	4	✓	
83960981	Start	ENSE00000945799	4	✓	***
83961072	End	ENSE00000945799	3	✓	
83961253	End	ENSE00002602349	1	-	
83965062	Start	ENSE00002581631	3	✓	***
83966331	End	ENSE00002581631	1	-	
83966332	End	ENSE00001391041	2	✓	

Supplementary Table ST4. Splice graph formation: Putative exons were selected using the designed priorities of the highest frequency at first phase, the lengths of exons on the second phase, and multiple exons including the intronic region in an equal condition of splice sites as the last phase.
*The multiple exons instead of the exon including intron region
**The longest exon (minimum start point for repeated end points)
***The splice sites with the highest frequency

Exon_Index	Position	Start/End
1	83949067	Start
	83949107	End
2	83949403	Start
	83949506	End
3	83953222	Start
	83953370	End
4	83957640	Start
	83957738	End
5	83959260	Start
	83959396	End
6	83960569	Start
	83960760	End
7	83960981	Start
	83961072	End
8	83965062	Start
	83966332	End

Supplementary Table ST5. Resulted splice graph has 8 putative exons.

Separate Supplemental Files:

File S4.xlsx: The data related to differentially expressed transcripts identification and gene ontology analysis of the example data for Chi square test

File S5.xlsx: Differentially expressed genes resulted from paclitaxel treatment on ovarian cancerous tissue

File S6.xlsx: Excel Data for testing

File S7.GTF: GTF Data for testing

File S8.GFF3: GFF3 Data for testing

File S9.xlsx: Data for statistical analysis testing
